# New PKS/NRPS Tenuazamines A–H from the Endophytic Fungus *Alternaria alternata* FL7 Isolated from *Huperzia serrata*

**DOI:** 10.3390/jof10120809

**Published:** 2024-11-21

**Authors:** Hao Zhang, Zhibin Zhang, Yiwen Xiao, Wen Wang, Boliang Gao, Yuhao Xie, Jiahao Xie, Xinhua Gao, Du Zhu

**Affiliations:** 1 Jiangxi Province Key Laboratory of Natural Microbial Medicine Research, Key Laboratory of Microbial Resources and Metabolism of Nanchang City, College of Life Sciences, Jiangxi Science and Technology Normal University, Nanchang 330013, China; 17609234825@163.com (H.Z.); wenwang_jiangxi@163.com (W.W.); gaoboliangspinach@aliyun.com (B.G.); hao18295767732@163.com (Y.X.); xiejiahao20010202@163.com (J.X.); gaoxinhuade@126.com (X.G.); 2 Jiangxi Province Key Laboratory of Biodiversity Conservation and Bioresource Utilization, School of Life Sciences, Jiangxi Normal University, Nanchang 330022, China; zzbbio@jxnu.edu.cn

**Keywords:** *Alternaria alternata*, *Huperzia serrata*, tenuazamine, tautomer, herbicides

## Abstract

In this paper, we present a novel class of hybrid polyketides, tenuazamines A–H (**1**–**8**), which exhibit a unique tautomeric equilibrium from *Alternaria alternata* FL7. The elucidation of the structures was achieved through a diverse combination of NMR, HR-ESIMS, and ECD methods, with a focus on extensive spectroscopic data analysis. Notably, compounds **1**, **4**, **8**–**9** exhibited potent toxic effects on the growth of *Arabidopsis thaliana*. This research expands the structural diversity of tenuazonic acid compounds derived from endophytic fungi and provides potential hit compounds for the development of herbicides.

## 1. Introduction

The genus *Alternaria alternata* (Fr.) Keissl., a member of the family *Pleosporaceae*, is known to exist as an endophyte, a parasite, or a saprophyte in various environments [1], such as soil, plants, air, and even some insects’ bodies [2,3,4,5]. *A. alternata* has been identified as a source of tenuazonic acid compounds (TeAs), alternariol (AOH), alternariol monomethyl ether (AME), altenuene (ALT), and tentoxin (TEN) [6]. Among these natural products, TeAs characterized by a pyrrolidine-2, 4-dione ring, are the most toxic (Figure S1, Supporting Information, SI) [7].

Tenuazonic acid (TeA, **9**, Figure S1, Supporting Information, SI) was first isolated in 1957 from the cultures of *A. tenuis* [8]. TeAs have been reported to be produced in other plant pathogenic fungi, such as *Ageratina adenophora*, *Stemphylium loti,* and *Epicoccum sorghinum* [9,10,11], with a broad spectrum of biological activities, including antitumor (TeA, **9**, Figure S1, Supporting Information, SI) [12], antifungal (cladodionen, **23**, Figure S1, Supporting Information, SI) [13], antiviral (cladosins C, **24**, Figure S1, Supporting Information, SI) [14], antibacterial and antibiotic (paecilosetin **16**, and altersetin **17,** Figure S1, Supporting Information, SI) [15,16], insecticidal, and phytotoxic activities (trichosetin, **27**, Figure S1, Supporting Information, SI) [17,18].

To the best of our knowledge, there are few reports about tautomer on the related secondary metabolites of *A. alternata* from endophytic fungus [19]. In this study, we considerably enriched the structural diversity of secondary metabolites of *A. alternata* and systematically determined its phytotoxicity in model plant, *Arabidopsis thaliana* (L.) Heynh. and providing potentially active compounds for eco-friendly weed control applications.

## 2. Materials and Methods

### 2.1. General Experimental Procedures

Electronic circular dichroism (ECD) spectra were measured using a Chirascan spectropolarimeter (Applied Photophysics, Leatherhead, UK). Column chromatography was performed using a 200–300-mesh silica gel (Qingdao Marine Chemical Co., Ltd., Qingdao, Shandong, China), a reversed-phase C18 gel (YMC Co., Ltd., Kyoto, Japan), and Sephadex LH-20 (GE Healthcare, Chicago, IL, USA). Semipreparative reversed-phase HPLC was performed using a Waters system (Milford, MA, USA) comprising a 2535 quaternary pump and a 2996 controller coupled to a 2489 dual-absorbance detector. UPLC-QToF-HRMS analyses were performed on ACQUITY H-Class UPLC (Waters system) coupled to a Xevo**^®^** G2-XS Q-TOF instrument (Waters system) with an ESI interface. All NMR spectra were acquired using an AVNEO 400-MHz NMR spectrometer (Bruker, Billerica, MA, USA). UV–vis spectroscopy was performed using a Lambda 365 instrument (PerkinElmer, Waltham, MA, USA). *Arabidospsis thaliana* was purchased from Zhengzhou Arabidopsis Biotechnology Co., Ltd. (Zhengzhou, Henan, China).

### 2.2. Molecular Identification of the Endophytic Fungi

The endophytic fungus FL7 was isolated from *H. serrata* at the Lushan Botanical Garden in Jiangxi Province and the Chinese Academy of Sciences [20]. The fungal DNA extraction and amplification were performed at the Jiangxi Province Key Laboratory of Natural Microbial Medicine Research using an F917891 Fungi Genomic Fungal DNA Kit (Macklin, Shanghai, China) and the PCR conditions as previously described (in 2019) [21]. Sequencing procedures were performed at the Shanghai Shenggong Bioengineering Technology Service Co., Ltd. (Shanghai, China). Molecular identification of the fungal endophyte was performed using (i) the primer pair ITS1 (5′-TCCGTAGGTGAACCTGCGG-3′) and ITS4 (5′-TCCTCCGCTTATTGATATATGC-3′) to amplify the 5.8S region of the ribosomal DNA and the two internal spacers ITS1 and ITS2 [22]. The sequence was subjected to the pairwise comparison using the BLASTN search tool with the Megablast algorithm on the NCBI platform. Top matching sequences were obtained considering similarity levels for species identification of 99.65% [21]. Therefore, it was identified as *A. alternata*. It was stored at the China Typical Culture Collection Center (http://cctcc.whu.edu.cn/) accesed on 24 October 2022 under the collection number CCTCC M 2023428.

### 2.3. Fermentation and Extraction

The endophytic fungus FL7 stored in an ultralow-temperature refrigerator at −80 °C was thawed, inoculated into Petri dishes containing PDA, and incubated for 7 d at 28 °C. Subsequently, mycelial hyphae were collected using an inoculation loop, transferred to Erlenmeyer flasks containing PDB, and shaken at 28 °C and 180 rpm/min for 4 d to prepare the seed solution. FL7 was inoculated in a solid brown rice medium in 500 × 1 L Erlenmeyer flasks, each containing 80 g of brown rice and 120 mL of tap water, and sterilized at 121 °C for 21 min in an autoclave.

After incubation at 28 °C for 30 days, the fermented material was extracted with MeOH (11 times, each for 24 h), and the solution was evaporated to dryness under reduced pressure to obtain an extract weighing 1.25 kg. The MeOH extract was mixed with an equal volume of distilled water to form a partial suspension and extracted thrice with an equal volume of petroleum ether and ethyl acetate (EtOAc) to obtain petroleum ether and ethyl acetate extracts (191 g). The EtOAc fraction was subjected to silica gel-based column chromatography with a stepwise-gradient elution using petroleum ether, petroleum-EtOAc, and EtOAc to yield four fractions 1–4. Fraction 4 (38 g) was further subjected to an MCI open-column chromatography using MeOH:H_2_O in a 5:95–100:0 gradient to obtain eight subfractions 4.1–4.8. Subfraction 4.3 (8.5 g) was subjected to silica gel-based column chromatography with a stepwise-gradient elution using CH_2_Cl_2_:MeOH to yield six super subfractions 4.3.1–4.3.6.

Super subfraction 4.3.1 (210 mg) was purified via semipreparative RP-HPLC using 35% (*v*/*v*) MeOH in H_2_O to afford **2** (17.5 mg; t_R_ = 9.2 min), **7** (1.1 mg; t_R_ = 13.0 min), **5** (11.4 mg; t_R_ = 15.0 min), and **1** (59.5 mg; t_R_ = 23.0 min). Super subfraction 4.3.2 (104 mg) was purified via semipreparative RP-HPLC using 10% MeOH in H_2_O to afford **3** (17.9 mg; t_R_ = 8.0 min), **4** (34.7 mg; t_R_ = 10.3 min), and **6** (2.8 mg, t_R_ = 14.0 min). Compound **8** was precipitated from the methanol solution of super subfraction 4.3.5 and obtained via recrystallization. Super subfraction 4.3.6 (145 mg) was purified through semipreparative RP-HPLC using 70% MeOH in H_2_O to afford **9** (41.2 mg; t_R_ = 9.1 min) and **10** (6.4 mg, t_R_ = 18.1 min).

Tenuazamine A (**1**): brown oil droplets; ^1^H and ^13^C data, Table 1 and Table 2; UV (500 µg/mL, MeOH) λmax (logε) 291.0 nm, Figure S7; HRESIMS *m/z* 197.1290 [M+H]^+^, (calcd. for C_10_H_17_N_2_O_2_, 197.1297); ${\left[\alpha \right]}_{D}^{25}$ −0.3 (*c* 2.0, MeOH).

Tenuazamine B (**2**): brown oil droplets; ^1^H and ^13^C data, Table 1 and Table 2; UV (500 µg/mL, MeOH) λ_max_ 298.1 nm and 302.1 nm, Figure S14; HRESIMS *m/z* 263.1359 [M+Na]^+^, (calcd. for C_12_H_20_N_2_O_3_Na, 263.1372); ${\left[\alpha \right]}_{D}^{25}$ −5.4 (*c* 1.0, MeOH).

Tenuazamine C (**3**): brown oil droplets; ^1^H and ^13^C data, Table 1 and Table 2; UV (500 µg/mL, MeOH) λ_max_ 304.1 nm, Figure S21; HRESIMS *m/z* 381.1633 [M+Na]^+^, (calcd. for C_16_H_26_N_2_O_7_Na, 381.1638); ${\left[\alpha \right]}_{D}^{25}$ 2.4 (*c* 1.0, MeOH).

Tenuazamine D (**4**): brown oil droplets; ^1^H and ^13^C data, Table 1 and Table 2; UV (500 µg/mL, MeOH) λ_max_ 302.9 nm, Figure S28; HRESIMS *m/z* 297.1802 [M+H]^+^, (calcd. for C_15_H_25_N_2_O_4_, 297.1814); ${\left[\alpha \right]}_{D}^{25}$ 0.25 (*c* 2.0, MeOH).

Tenuazamine E (**5**): brown oil droplets; ^1^H and ^13^C data, Table 2 and Table 3; UV (500 µg/mL, MeOH) λ_max_ 287.4 nm, Figure S35; HRESIMS *m/z* 211.1092 [M−H]^−^, (calcd. for C_10_H_15_N_2_O_3_, 211.1083); ${\left[\alpha \right]}_{D}^{25}$ 5.9 (*c* 1.0, MeOH).

Tenuazamine F (**6**): brown oil droplets; ^1^H and ^13^C data, Table 2 and Table 3; UV (500 µg/mL, MeOH) λ_max_ 291.0 nm, Figure S42; HRESIMS *m/z* 211.1092 [M−H]^−^, (calcd. for C_11_H_14_N_2_O_4_, 211.1083); ${\left[\alpha \right]}_{D}^{25}$ −0.6 (*c* 2.0, MeOH).

Tenuazamine G (**7**): brown oil droplets; ^1^H and ^13^C data, Table 2 and Table 3; UV (500 µg/mL, MeOH) λ_max_ 291.0 nm, Figure S51; HRESIMS *m/z* 183.1116 [M+H]^+^, (calcd. for C_9_H_15_N_3_O_3_, 183.1133); ${\left[\alpha \right]}_{D}^{25}$ −8.05 (*c* 2.0, MeOH).

Tenuazamine H (**8**): brown oil droplets; ^1^H and ^13^C data, Table 2 and Table 3; HRESIMS *m/z* 282.1800 [M+H]^+^, (calcd. for C_14_H_24_N_3_O_3_, 282.1812); ${\left[\alpha \right]}_{D}^{25}$ 5.1 (*c* 2.0, MeOH).

### 2.4. Calculation Part

Conformational analyses were conducted through random searches by employing the Sybyl-X 2.0 program using the MMFF94S force field with an energy cutoff of 5 kcal/mol [23]. Results revealed eight low-energy conformers. Subsequently, geometry optimizations and frequency analyses were performed at the B3LYP-D3(BJ)/6-31G* level in CPCM methanol using ORCA5.0.1. [24]. All conformers used for the property calculations in this work were characterized to be stable points on the potential energy surface without any imaginary frequencies. The excitation energies, oscillator strengths, and rotational strengths (velocity) of the first 60 excited states were calculated using the time-dependent density functional theory (TD-DFT) methodology at the PBE0/def 2-TZVP level with CPCM methanol using ORCA5.0.1 [24]. The ECD spectra were simulated using the overlapping Gaussian function (half of the bandwidth was at the 1/e peak height; sigma = 0.30 for all) [25]. Gibbs free energies of conformers were determined via thermal correction at the B3LYP-D3(BJ)/6-31G* level, and electronic energies were evaluated at the wB97M-V/def 2-TZVP level with CPCM methanol using ORCA5.0.1 [24]. To obtain the final spectra, averages of the simulated conformer spectra were calculated according to the Boltzmann distribution theory and their relative Gibbs free energy. The absolute configuration of the only chiral center was determined by comparing the experimentally obtained spectra with the theoretically calculated ones.

### 2.5. Arabidospsis thaliana Phytoxicity Assays

The activity was determined according to the method described by Shi et al. [26]. All experiments were performed on *A. thaliana* leaves of the Columbia-0 ecotype 441 (Col-0) at 3 weeks old and were adapted for 24 h at 22 °C ± 0.5 °C in a growth chamber prior to inoculation. By taking 5 µL of a solution containing compounds **1**–**9,** glyphosate (dissolved in methanol, 1 mg/mL), and methanol, and dropping it onto the surface of *Arabidopsis* leaves, all leaves in each plant participated in the test. Glyphosate and methanol as positive and negative control groups, respectively, were used to compare the changes in *Arabidopsis* leaves in the experimental group. After adding the medication dropwise, it was placed in a light incubator (22 °C, humidity of 70%, illumination of 2000–3000 lux, photoperiod 14 h/8 h) and observed for 24 h for 7 times.

## 3. Results

### 3.1. Structure Elucidation

**Figure 1:**
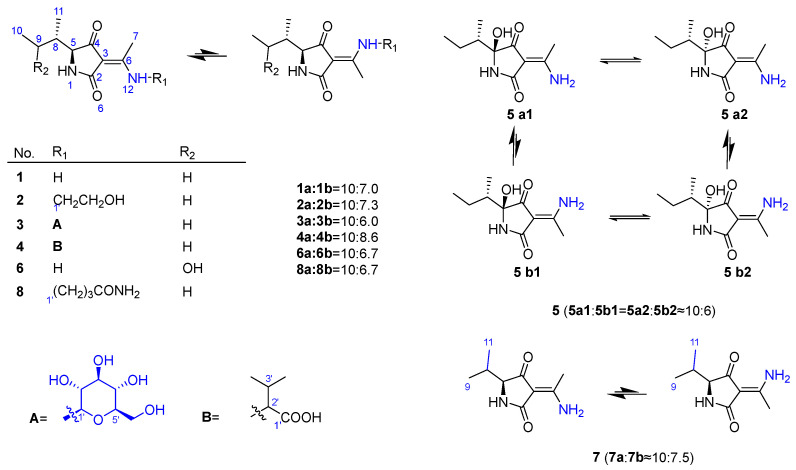
Chemical structures of **1**–**8** derived from *A. alternata* FL7.

**Figure 2:**
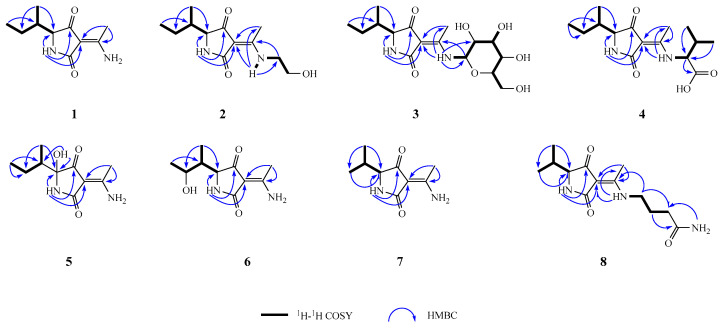
The key COSY (bond lines) and HMBC (blue arrows) correlations of compounds **1**–**8**.

**Figure 3:**
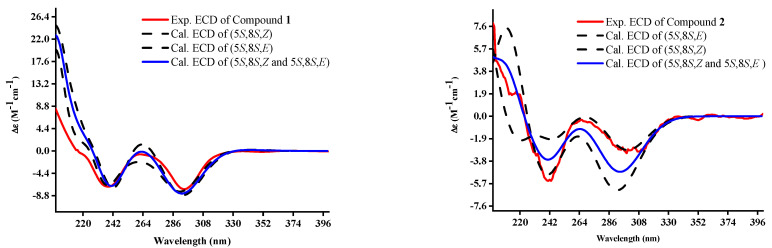
Experimental and calculated ECD spectra of compounds **1**–**2**.

Compound **1** was obtained as brown oil droplets and had the molecular formula C_10_H_16_N_2_O_2_, as determined by the protonated ion peak at *m/z* 197.1290 in the (+)-HRMS(ESI) spectrum ([M+H]^+^, calcd. 197.1297), accounting for four indices of hydrogen deficiency. The NMR spectrum showed signal pairs with different intensities and the reproducibility for tautomers **1a** and **1b** in HPLC analysis of the rejections of tautomers **1a** or **1b**, respectively (Figures S2 and S7, SI), suggesting tautomerism for compound **1**. To facilitate structural analysis, we preferentially used the NMR signals of the predominant tautomer (**1a**) for the structural elucidation. The ^1^H NMR spectrum of tautomer **1a** (Table 1) in DMSO-*d*_6_ gave three singlet signals at *δ*_H_ 8.64, 9.42 (each 1H for 12-NH_2_), and *δ*_H_ 7.53 (1H for 1-NH), a nitrogen-bearing methine signal resonating at *δ*_H_ 3.44 (1H, br d, *J* = 2.9 Hz, H-5), as well as one olefinic methyl at *δ*_H_ 2.30 (3H, s, H-7), and two aliphatic methyl signals at *δ*_H_ 0.79 (3H, t, *J* = 7.4 Hz) and 0.88 (3H, d, *J* = 7.0 Hz) in the high-field region (Figure 1). The ^13^C NMR spectrum showed 10 carbon signals, including signals assigned to two carbonyl groups (C-2, *δ*_C_ 174.6 and C-4, *δ*_C_ 195.9), two olefinic carbons at *δ*_C_ 167.2 (C-6) and 95.4 (C-3), one N-bearing methine at *δ*_C_ 64.9 (CH-5), one sp^3^ methylene (CH_2_-9, *δ*_C_ 23.0), one sp^3^ methine (CH-8, *δ*_C_ 36.5), and three methyls (*δ*_C_ 18.5, 15.8, 11.9) in the high-field region. Two carbonyl groups and one carbon–carbon double bond occupied three degrees of unsaturation, revealing a monocyclic system in tautomer **1a**. Further analysis of the ^1^H–^1^H COSY spectrum revealed the presence of a *sec*-butyl moiety by the correlation systems from H_3_-10 (*δ*_H_ 0.79) to H_3_-11 (*δ*_H_ 0.88) via H_2_-9 (*δ*_H_ 1.08 and 1.23) and H-8 (*δ*_H_ 1.72) (Figure 2). The aforementioned NMR data indicated tautomer **1a** to be a structural analog of TeA (**9**), with the differences being the 3-NH_2_ substituted *α, β*-unsaturated keto in tautomer **1a** rather than the corresponding 1-OH substituted *α, β*-unsaturated keto in the head-to-tail transposition position [26,27]. This was further verified through HMBC analysis, especially by the cross-peaks from H_3_-7 (*δ*_H_ 2.30) to C-3 (*δ*_C_ 95.4) and C-6 (*δ*_C_ 167.2) and H-1 (*δ*_H_ 7.53) to C-3 and C-4 (*δ*_C_ 195.9) (Figure 2). The loose end of C-6 was confirmed to be the 6-NH_2_ group by the chemical shifts for C-3 and C-6 due to the electron-donating inductive effect of the remaining amino group. This was reasonably in accordance with the elementary composition for tautomer **1a**. The relative configurations of the carbon centers for tautomer **1** were proposed as depicted on the basis of chemical evidence and biogenetic considerations which were consistent with those of tenuazonic acid with a Δ*δ*_C_ value within 0.9–1.6 ppm, especially the stereo centers of C-5 (Δ*δ*_C_ 0.9–1.4 ppm) and C-8 (Δ*δ*_C_ 1.1–1.6 ppm) [28]. The minor tautomer (**1b**) was ascribed to be the same gross structure as that of tautomer **1a** by the compressive assignments of the NMR data, especially the scrutiny of the 2D NMR data of tautomer **1b,** and the spectra were examined for evidence of minor tautomer and assigned their peaks, where possible (Table 1). Compared to the major tautomer **1a**, the intramolecular hydrogens bond formation for **1b** between 12-NH_2_ with C-4 rather than between 12-NH_2_ with C-2 were confirmed by the down-fielded shifts for C-4 (Δ*δ*_C_ +1.6 ppm) and up-fielded shifts for C-2 (Δ*δ*_C_ −2.5 ppm) [27,28], suggesting the *Z/E* isomerism for carbon–carbon double bonds between the predominant tautomer (**1a**) and the minor one (**1b**). To determine the absolute configuration of compound **1**, we calculated the ECD spectra of tautomers **1a** and **1b** as the same ratio of 10:7 (same as identified by ^1^H NMR data) using TD-DFT at the B3LYP D3(BJ)/6-31G level with the CPCM model [24]. Fortunately, the calculated spectra of **1** 5*S*, 8*S*, *Z* (**1a**) and 5*S*, 8*S*, *E* (**1b**) = 10:7 were in good agreement with the corresponding experimental spectrum of compound **1** (Figure 3). Thus, the structure of compound **1** was established, as shown in Figure 1, and named tenuazamine A.

Compound **2** was also obtained as brown oil droplets and had the molecular formula C_12_H_20_N_2_O_3_, as indicated by the positive ion peak at *m/z* 263.1359 in the (+)-HRMS(ESI) spectrum ([M+Na]^+^, calcd. 263.1372). The ^1^H and ^13^C NMR spectra of tautomer **2a** resembled these of the predominant tautomer **1a** in compound **1** (Table 1 and Table 2), with the major differences being the presence of the two additional methylene signals at *δ*_C_ 59.7 (t, C-14) and 44.7 (t, C-13). Considering the molecular formula of **2a**, a hydroxyethyl group has to be substituted on the amino group of C-6. Hence, the resulting structure was further verified using detailed ^1^H–^1^H COSY correlations between H-1′ (*δ*_H_ 3.40) and NH-12 (*δ*_H_ 10.58), together with the cross-peaks from H_2_-1′ to C-6 (*δ*_C_ 167.9), and NH-12 to C-1′ (*δ*_C_ 44.7) in the HMBC spectrum (Figure 2), and the up-fielded shift for CH_3_-7 (*δ*_C_ −4.6 ppm) in ^13^C NMR data. Similar to compound **1**, the *Z/E* isomerism of carbon–carbon double bonds between the predominant tautomer (**2a**) and the minor one (**2b**) in compound **2**, was confirmed by the down-fielded shifts for C-4 (Δ*δ*_C_ +2.8 ppm) and up-fielded shifts for C-2 (Δ*δ*_C_ −2.9 ppm) for **2b**, [28] due to the intramolecular hydrogens bond formation for **2b** between 12-NH with C-4 rather than between 12-NH with C-2. The relative configurations of compound **2** were consistent with those of **1**, as determined by a comparison of the NMR shifts of the *sec*-butyl moieties, especially the ^13^C NMR data of the center carbons C-5 (Δ*δ*_C_ −0.3 ppm) and C-8 (Δ*δ*_C_ +0.2 ppm) and biogenetic considerations. The absolute configurations of **2** were assigned as (5*S*, 8*S*, *Z* for **2a** and 5*S*, 8*S*, *E* for **2b**) by comparing its experimental and calculated ECD spectra (Figure 3). Therefore, the structure of compound **2** was established, as shown in Figure 1, and it was named tenuazamine B.

Compound **3** was also obtained as brown oil droplets and had the molecular formula C_16_H_26_N_2_O_7_, as gleaned from the sodiated molecular ion peak at *m/z* 381.1633 in the (+)-HRMS(ESI) analysis ([M+Na]^+^, calcd. 381.1638), with one more degree of unsaturation than compound **1**. The NMR data of six additional oxygenated ones (five methines and one methylene) in compound **3** indicated that it was a glycoside derivative of **1**, and the NMR signals of the notable tautomer (**3a**) were used for the subsequent structural interpretation. Except for six additional oxygenated carbons (*δ*_C_ 81.7, 78.8, 77.1, 73.4, 69.8, 60.9), the 1D NMR data (Table 1 and Table 2) of **3a** exhibited similarities to those of **1a**, suggesting the structural resemblance between **3a** and **1a**. Besides the difference at C-7 (Δ*δ*_C_ −5.0 ppm), all other carbons with almost identical chemical shifts (Δ*δ*_C_ 0.0–0.5 ppm), that suggested the glycoside appendage was attached to the 12-amino group via the anomeric C-1′ (*δ*_C_ 81.7) in **3a**. This structural deduction was confirmed by careful interpretation of the 2D NMR data, especially, the HMBC correlations from H-1′ (*δ*_H_ 4.69) to C-6 (*δ*_C_ 167.7) and from NH-12 (*δ*_H_ 10.73) to C-1′ (*δ*_C_ 81.7) and C-2′ (*δ*_C_ 73.4) (Figure 2), as well as the ^1^H–^1^H COSY correlation systems from N-12 to H_2_-6′ (Figure 2). The relative configuration of the aglycone unit in **3a** was deduced to be consistent with that of **1a** by comparing the NMR shifts of the stereocenters with almost the same chemical carbon shift values for C-5, C-8, C-9, C-10, and C-11 for **1a** and **3a**. Furthermore, the relative configuration of the 1-amino-1-deoxy-*β*-D-glucose was ascribed by the multiplicities of the coupling constants for *J*_1′,2′_, *J*_2′,3′_, *J*_3′,4′_, *J*_4′,5′_ with about 9.0 Hz, which was supported by biogenetic considerations. Similar to compound **1**, the *Z/E* isomerism of carbon–carbon double bonds between the predominant tautomer (**3a**) and the minor one (**3b**) in compound **3**, based on the down-fielded shifts for C-4 (*δ*_C_ +2.8 ppm) and up-fielded shifts for C-2 (*δ*_C_ −3.1 ppm) for **3b** [28] due to the intramolecular hydrogens bond formation for **3b** between 12-NH with the keto carbonyl (C-4) rather than between 12-NH with the amidocarbonyl group (C-2). Finally, the chemical structure of the new *N*-glycoside was established, and it was named tenuazamine C.

Compound **4** was obtained as brown oil droplets and had the molecular formula C_15_H_24_N_2_O_4_, as indicated by the *quasi*-molecular ion peak of *m/z* 297.1802 in the (+)-HRMS(ESI) spectrum (calcd. 297.1814), accounting for five degrees of unsaturation. The ^13^C NMR spectrum (Table 1 and Table 2) included 15 carbon signals, with 10 being considerably similar to those of tenuazamine A (**1**), indicating that it was a structural congener of compound **1**. The noticeable difference was the presence of five additional carbon signals, one carbonyl (*δ*_C_ 172.0), two methines (*δ*_C_ 63.2 and 31.4), and two methyls (*δ*_C_ 19.4 and 18.1) in **4**. Considering the molecular formula and through the analysis of the obtained 2D NMR spectra (Figure 2), the new signals were assigned to a 3-methylbutanoic acid moiety using the HMBC correlations from H-2′ (*δ*_H_ 3.76) to C-1′ (*δ*_C_ 172.0) and the cross-peaks between H-3′ (*δ*_H_ 2.15) and H_3_-4′ (*δ*_H_ 0.86) and H_3_-5′ (*δ*_H_ 0.91) in the ^1^H–^1^H-COSY spectrum of the major tautomer **4a**. Furthermore, the ^1^H–^1^H-COSY correlation (Figure 1) between 12-NH and H-2′ revealed the association of the 3-methylbutanoic acid moiety, to be a five-carbon adduct of the 12-NH. Similar to compound **1**, the *Z/E* isomerism of carbon–carbon double bonds between the predominant tautomer (**4a**) and the minor one (**4b**) in compound **4**, was confirmed by the down-fielded shifts for C-4 (+2.8 ppm) and up-fielded shifts for C-2 (−2.8 ppm) for **4b** [28] due to the intramolecular hydrogens bond formation for **4b** between 12-NH with keto group (C-4) rather than between 12-NH with acylamino (C-2). Finally, the chemical structure of the new compound was established, as shown in Figure 1, and it was named tenuazamine D.

Compound **5** was obtained as brown oil droplets and had the molecular formula of C_10_H_16_N_2_O_3_, as indicated by the negative *quasi*-molecular ion peak at *m/z* 211.1092 [M−H]^−^ (calcd. 211.1083) in (−)-HRMS(ESI). Its 1D NMR data (Table 2 and Table 3) were highly similar to those of **1**, but with the absence of the proton signal of CH-5 (*δ*_H_ 3.44/3.53 and *δ*_C_ 64.9/63.6 for **1a** and **1b**), and in the concomitant presence of non-hydrogen bearing carbon resonances (*δ*_C_ 87.85/87.87 for **5a1** and **5a2**, 87.02/87.03 for **5b1** and **5b2**, respectively), suggesting the existence of a new substituent at C-5, which was then identified as hydroxyl groups by ESIMS and HMBC spectra (Figure 2). Specifically, the HMBC correlations (Figure 2) from the 1-NH signals at *δ*_H_ 7.70/7.77 (1H, s, 1-NH), 5.60/5.62 (1H, s, 5-OH), and 0.61/0.89 (3H, d, *J* = 6.9 Hz, H_3_-11) to the non-oxygenated bearing C-5 (*δ*_C_ 87.9) confirmed that the hydroxyl groups were substituted at C-5 for tautomers **5a1** and **5a2**. Given the hemiketal property of the hydroxyl group, the configurations of C-5 were rapidly isomerized, and the isomers existed in the solution in nearly equal quantities. As compared with those of **1a**, the relative *α*-configurations for 5-OHs of tautomers **5a2** and **5b2** were assigned by the up-fielded ^13^C NMR data for C-8, C-9, and C-11 with more values of Δ*δ*_C_ −1.5, and −4.0 ppm by the *γ*-gauche effects resulting from the 5*α*-OHs. Consequently, the 5-OHs of tautomers **5a1** and **5b1** were *β*-oriented with less values of Δ*δ*_C_ −2.6 ppm for C-11, even down-fielded shift for C-9 (Δ*δ*_C_ +0.3 ppm) resulting in less repulsion of 5-NHs. Similar to compound **1**, the *Z/E* isomerism of carbon–carbon double bonds between the predominant tautomers (**5a1** and **5a2**) and the minor ones (**5b1** and **5b2**) in compound **5** was verified by the down-fielded shifts for C-4 (Δ*δ*_C_ +2.3 ppm) and up-fielded shifts for C-2 (Δ*δ*_C_ −2.7 ppm) for **5b1** and **5b2**. This was due to the intramolecular hydrogen bond formation for **5b1** and **5b2** between 12-NH with keto functional groups (C-4) rather than between 12-NH with amide carbonyls (C-2). Thus, the chemical structure of the new compound was established, as shown in Figure 1, and it was named tenuazamine E.

Compound **6** was obtained as brown oil droplets and had the molecular formula C_10_H_16_N_2_O_3_, as indicated by the negative HR-ESI–MS (*m/z* based on [M−H]^−^: 211.1092; calcd. 211.1083), suggesting to be an oxygenated derivative of compound **1**. The ^13^C NMR spectrum (Table 2) of the major tautomer **6a** shows 10 carbon signals being considerably similar to those of the tautomer **1a** of tenuazamine A (**1**), being the absence of a methene (*δ*_H_ 1.08, 1.23, and *δ*_C_ 23.0) and the concomitant presence of an oxygen-bearing methine (*δ*_H_ 3.61 and *δ*_C_ 68.5) in compound **6a**, as well as the down-field chemical shifts of CH_3_-10 (Δ*δ*_H_ +0.27 ppm and Δ*δ*_C_ +9.6 ppm) and CH-8 (*δ*_H_ +0.05 ppm and *δ*_C_ +4.8 ppm) by the inductive effect of hydroxyl group and up-field chemical shifts for CH_3_-11 (Δ*δ*_H_ −0.27 ppm and Δ*δ*_C_ −7.5 ppm) due to the *γ*-gauche effect between CH_3_-11 and 9-OH. The structural assignment of **6a** was confirmed by the interpretation of its 2D NMR data, especially the HMBC cross-peaks from H_3_-11 (*δ*_H_ 0.61) to CH-9 (*δ*_C_ 68.5) and the spin-coupling systems from H-8 (*δ*_H_ 1.77) to H_3_-11 (*δ*_H_ 0.61), and from H-5 (*δ*_H_ 3.60) to H_3_-10 (*δ*_H_ 1.06) via H-9 (*δ*_H_ 3.61) ascribed from the ^1^H-^1^H COSY spectrum. Similar to compound **1**, the *Z/E* isomerism of carbon–carbon double bonds between the predominant tautomer (**6a**) and the minor one (**6b**) in compound **6** was verified by the down-fielded shifts for C-4 (Δ*δ*_C_ +2.6 ppm) and up-fielded shifts for C-2 (Δ*δ*_C_ −2.5 ppm) for **6b**, due to the intramolecular hydrogens bond formation for **6b** between 12-NH with keto functional groups (C-4) rather than between 12-NH with amide carbonyls (C-2). Finally, the chemical structure of the new compound **6** was established (Figure 1), and it was named tenuazamine F.

Compound **7** was obtained as brown oil droplets and had the molecular formula C_9_H_14_N_2_O_2_, as indicated by positive HR-ESI–MS (*m/z* based on [M+H]^+^: 183.1116; calcd. 183.1133), with less CH_2_ moiety than compound **1**. The ^1^H and ^13^C NMR spectra of the major tautomer **7a** resembled those of tautomer **1a** (Table 2 and Table 3), with the major differences being one less methene signal and the presence of a doublet methyl group (CH_3_-9, *δ*_C_ 19.5, *δ*_H_ 0.92) in **7a** instead of the corresponding triplet methyl moiety (CH_3_-10, *δ*_C_ 11.9, *δ*_H_ 0.79) in **1a**. This structural deduction was confirmed by the 2D NMR data, especially by the HMBC correlations from H_3_-11 (*δ*_H_ 0.67) to CH_3_-9 (*δ*_C_ 19.5) and C-5 (*δ*_C_ 65.1), as well as the spin-coupling systems from H-8 to H_3_-9 and H_3_-11 assigned by the ^1^H-^1^H COSY spectrum. Similar to compound **1**, the *Z/E* isomerism of carbon–carbon double bonds between the predominant tautomer (**7a**) and the minor one (**7b**) in compound **7** was verified by the down-fielded shifts for C-4 (Δ*δ*_C_ +2.6 ppm) and up-fielded shifts for C-2 (Δ*δ*_C_ −2.5 ppm) for **7b**,^19^ due to the intramolecular hydrogens bond formation for **7b** between 12-NH with keto functional groups (C-4) rather than between 12-NH with amide carbonyls (C-2). Finally, the chemical structure of compound **7** was established, as shown in Figure 1, and it was named tenuazamine G.

Compound **8** was a white amorphous powder and had the molecular formula of C_14_H_23_N_3_O_3_, as indicated by the positive HR-ESI–MS (*m/z* [M+H]^+^: 282.1800, calcd. 282.1812), accounting for five degrees of unsaturation. The ^1^H and ^13^C NMR spectra of major tautomer **8a** resembled these of the predominant tautomer **1a** in compound **1** (Table 2 and Table 3), with the major differences being the presence of the four extra carbon signals for one amido-carbonyl carbon (*δ*_C_ 173.3) and three aliphatic methylene carbons (*δ*_C_ 41.4, 31.8, and 25.0) in **8a**. Considering the elementary composition of **8a**, 4-butyramide moiety has to be substituted on the 12-NH group. Hence, the resulting structure was further verified using detailed ^1^H–^1^H COSY correlations from 3′-CH_2_ (*δ*_H_ 1.74) to 12-NH (*δ*_H_ 10.53), together with the cross-peaks from H_2_-1′ (*δ*_H_ 3.33) to C-6 (*δ*_C_ 167.3), and H_2_-2′ (*δ*_H_ 1.74) to C-4′ (*δ*_C_ 173.3), and 4′-NH_2_ (*δ*_H_ 6.82, 7.37) to CH_2_-3′ (*δ*_C_ 31.8) in the HMBC spectrum (Figure 2). Similar to compound **1**, the *Z/E* isomerism of carbon–carbon double bonds between the predominant tautomer (**8a**) and the minor one (**8b**) in compound **8**, was confirmed by the down-fielded shifts for C-4 (Δ*δ*_C_ +3.1 ppm) and up-fielded shifts for C-2 (Δ*δ*_C_ −3.3 ppm) for **8b**, due to the intramolecular hydrogens bond formation for **8b** between 12-NH with keto carbonyl rather than between 12-NH with amide carbonyl. The relative configurations of compound **8** were consistent with those of **1**, as determined by a comparison of the NMR shifts of the *sec*-butyl moieties, especially the ^13^C NMR data of the center carbons C-5 (Δ*δ*_C_ +0.04 ppm) and C-8 (Δ*δ*_C_ −0.03 ppm) and biogenetic considerations. Finally, the structure of **8** was established, as shown in Figure 1. and it was named tenuazamine H.

### 3.2. Biological Activity of the Compounds on A. thaliana

**Figure 4:**
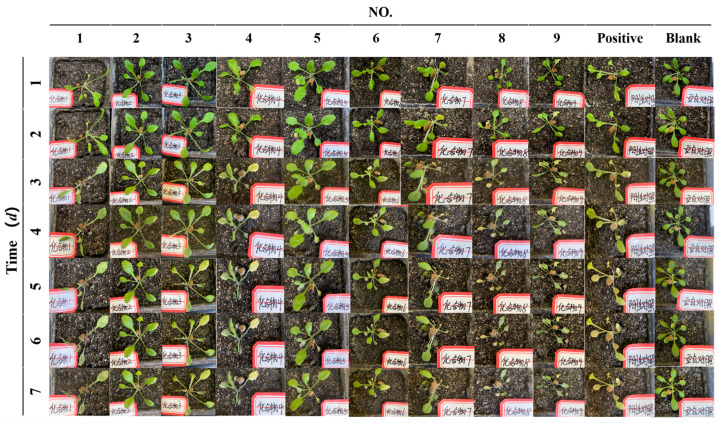
Toxicity of compounds **1**–**9** to *Arabidopsis thaliana*. (Note. the Chinese characters in the picture means “compound”).

The activity results indicate that all tested substances (**1**–**9**) displayed varying levels of phytotoxicity, as depicted in Figure 4. Evaluate the effects of various compounds on Arabidopsis growth by observing plant height, leaf quantity, color, and spots. Notably, the blank control group of *A. thaliana* did not receive any impact. Tenuazamine A (**1**), tenuazamine D (**4**), tenuazamine H (**8**), TeA (**9**), and the positive control (glyphosate) also exhibited obvious toxicity in the leaves of *A. thaliana* because they noticeably turned yellow (Figure 4). In addition, the *Arabidopsis* plants tested for the above compounds did not show significant changes in plant height, spots, or quantity, except for leaf chlorosis. Shi et al. uncovered that TeA toxin-induced photosynthetically generated 1O_2_ in *A. thaliana* triggers EXECUTER1 (EX1)/EX2-mediated chloroplast-to-nucleus retrograde signaling (RS), ultimately leading to the demise of *A. thaliana* leaves [29]. As depicted in Figure 4, Compared to compound **1**, the decrease in activity observed in compounds **2**–**3** and **5**–**7** could be attributed to the varying substitutions on the 12-NH_2_ moiety, but the butanamide substituted congener **8** with more potent activity indicated the butanamide group played a key role to the herbicidal activity. The tested compounds with hydroxyl groups on the core skeletons, such as hydroxyls on C-5 (**5**) and C-9 (**6**), were nearly inactive. Furthermore, compound **7** with one carbon less showed less toxicity and indicated the more compact carbon skeleton attenuated the herbicidal activity. This phenomenon may be linked to the attenuation strategies adopted by pathogenic fungi transitioning into endophytic fungi, as they adapt to the less hostile plant endosphere environment.

## 4. Conclusions

These results considerably enrich the secondary metabolite library of *A. alternata* and provide a series of new compounds with potentially phytotoxic activity on *Arabidopsis thaliana*. Meanwhile, they may have the potential for further research as biological herbicides, providing reference value for the secondary metabolites of fungi in agricultural weed control work.

## Figures and Tables

**Table 1:** ^1^H NMR Spectroscopic Data for Compounds **1**–**4** in DMSO-*d*_6_.

No.	1a	1b	2a	2b	3a	3b	4a	4b
**5**	3.44 (1H, br d, 2.9)	3.53 (1H, br d, 2.9)	3.44 (1H, br d, 2.9)	3.52 (1H, overlapped)	3.51 (1H, br d, 2.9)	3.60 (1H, br d, 3.2)	3.41 (1H, overlapped)	3.49 (1H, br d, 3.2)
**7**	2.30 (3H, s)	2.33 (3H, s)	2.45 (3H, s)	2.47 (3H, s)	2.52 (3H, s)	2.57 (3H, s)	2.37 (3H, s)	2.40 (3H, s)
**8**	1.72 (1H, m)	1.72 (1H, m)	1.72 (1H, m)	1.72 (1H, m)	1.74 (1H, m)	1.74 (1H, m)	1.72 (1H, m)	1.72 (1H, m)
**9**	1.08, 1.23 (each 1H, m)	1.08, 1.23 (each 1H, m)	1.05, 1.21 (each 1H, m)	1.05, 1.21 (each 1H, m)	1.09, 1.23 (each 1H, m)	1.09, 1.23 (each 1H, m)	1.08, 1.24 (each 1H, m)	1.08, 1.24 (each 1H, m)
**10**	0.79 (3H, t, 7.4)	0.79 (3H, t, 7.4)	0.77 (3H, t, 7.4)	0.78 (3H, t, 7.4)	0.80 (3H, t, 7.4)	0.80 (3H, t, 7.4)	0.80 (3H, t, 7.4) ^a^	0.79 (3H, t, 7.4) ^a^
**11**	0.88 (3H, d, 7.0)	0.87 (3H, d, 6.9)	0.87 (3H, d, 7.0)	0.86 (3H, d, 6.9)	0.89 (3H, d, 6.9)	0.88 (3H, d, 6.9)	0.89 (3H, d, 6.9)	0.88 (3H, d, 6.9)
**1′**			3.40 (2H, m)	3.40 (2H, m)	4.69 (1H, dd, 8.7, 8.1)	4.75 (1H, dd, 8.8, 7.9)		
**2′**			3.55 (2H, m)	3.55 (2H, m)	3.07 (1H, t-like, 8.8)	3.08 (1H, t-like, 8.8)	3.76 (1H, dd, 9.0, 4.4)	3.82 (1H, dd, 9.0, 4.2)
**3′**					3.25 (1H, t-like, 9.0)	3.26 (1H, t-like, 9.0)	2.15 (1H, m)	2.13 (1H, m)
**4′**					3.09 (1H, t-like, 9.5)	3.09 (1H, t-like, 9.5)	0.86 (1H, d, 6.6)	0.86 (1H, d, 6.6)
**5′**					3.27 (1H, m)	3.27 (1H, m)	0.91 (1H, d, 6.6)	0.91 (1H, d, 6.6)
**6′**					3.42, 3.66 (each 1H, m)	3.42, 3.66 (each 1H, m)		
**1-NH**	7.53 (1H, br s)	7.31 (1H, br s)	7.55 (1H, br s)	7.29 (1H, br s)	7.84 (1H, s)	7.55 (1H, s)	7.46 (1H, s)	7.22 (1H, s)
**12-NH**			10.58 (1H, t, 5.7)	10.89 (1H, t, 5.6)	10.73 (1H, d, 8.1)	10.91 (1H, d, 7.9)	10.80 (1H, d, 9.0)	11.12 (1H, d, 9.0)
**NH_2_**	8.64, 9.42 (each 1H, br s)	8.78, 9.67 (each 1H, br s)						

^a^ Interchangeable.

**Table 2:** ^13^C NMR Spectroscopic Data for Compounds **1**–**8** in DMSO-*d*_6_.

No.	1a	1b	2a	2b	3a	3b	4a	4b	5a1–2	5b1–2	6a	6b	7a	7b	8a	8b
**2**	174.6 (s)	172.1 (s)	175.5 (s)	172.6 (s)	174.4 (s)	171.3 (s)	175.5 (s)	172.7 (s)	173.8/173.9 (s)	171.1/171.2 (s)	174.7 (s)	172.2 (s)	174.7 (s)	172.2 (s)	175.3 (s)	172.0 (s)
**3**	95.4 (s)	97.0 (s)	95.3 (s)	97.1 (s)	96.6 (s)	98.2 (s)	94.7 (s)	96.5 (s)	93.5/93.6 (s)	95.1/95.2 (s)	94.9 (s)	96.5 (s)	95.2 (s)	96.8 (s)	95.0 (s)	97.0 (s)
**4**	195.9 (s)	198.5 (s)	195.7 (s)	198.5 (s)	196.2 (s)	199.4 (s)	194.9 (s)	197.5 (s)	196.3/196.4 (s)	198.6/198.7 (s)	196.0 (s)	198.6 (s)	195.8 (s)	198.4 (s)	195.2 (s)	198.3 (s)
**5**	64.9 (d)	63.6 (d)	65.2 (d)	63.3 (d)	65.0 (d)	63.1 (d)	64.8 (d)	63.0 (d)	87.9/87.9 (s)	87.0/87.0 (s)	62.5 (d)	61.3 (d)	65.1 (d)	63.9 (d)	64.9 (d)	63.0 (d)
**6**	167.2 (s)	167.3 (s)	167.9 (s)	168.1 (s)	167.7 (s)	168.0 (s)	166.0 (s)	165.6 (s)	166.8 (s)	166.9/167.0 (s)	167.4 (s)	167.4 (s)	167.3 (s)	167.4 (s)	167.3 (s)	167.6 (s)
**7**	18.5 (q)	17.6 (q)	13.9 (q)	13.2 (q)	13.5 (q)	12.7 (q)	13.9 (q)	13.1 (q)	18.4/18.4 (q)	17.7/17.7 (q)	18.5 (q)	17.6 (q)	18.5 (q)	17.6 (q)	13.3 (q)	12.5 (q)
**8**	36.5 (d)	36.6 (d)	36.7 (d)	36.8 (d)	36.5 (d)	36.6 (d)	36.5 (d)	36.6 (d)	*	*	41.3 (d)	41.2 (d)	29.5 (d)	29.6 (d)	36.5 (d)	36.6 (d)
**9**	23.0 (t)	23.3 (t)	23.1 (t)	23.4 (t)	23.0 (t)	23.3 (t)	22.9 (t)	23.2 (t)	21.5/23.3 (t)	21.6/23.4 (t)	68.5 (d)	68.4 (d)	19.5 (q)	19.2 (q)	22.9 (t)	23.2 (t)
**10**	11.9 (q)	11.9 (q)	12.2 (q)	12.1 (q)	11.9 (q)	11.8 (q)	11.9 (q)	11.9 (q)	11.9/12.2 (q)	11.9/12.2 (q)	21.5 (q)	21.5 (q)	15.6 (q)	15.8 (q)	11.9 (q)	11.8 (q)
**11**	15.8 (q)	15.6 (q)	16.1 (q)	15.8 (q)	15.8 (q)	15.5 (q)	16.0 (q)	15.6 (q)	11.8/13.2 (q)	11.9/13.2 (q)	8.3 (q)	8.3 (q)			15.9 (q)	15.6 (q)
**1′**			44.7 (t)	44.9 (t)	81.7 (d)	81.9 (d)	172.0 (s)	171.8 (s)							41.4 (t)	41.6 (t)
**2′**			59.7 (t)	59.6 (t)	73.4 (d)	73.5 (d)	63.2 (d)	63.2 (d)							25.0 (t)	24.9 (t)
**3′**					77.1 (d)	77.0 (d)	31.4 (d)	31.2 (d)							31.8 (t)	31.7 (t)
**4′**					69.8 (d)	69.8 (d)	19.4 (q) ^b^	19.5 (q) ^b^							173.3 (s)	173.3 (s)
**5′**					78.8 (d)	78.9 (d)	18.1 (q)	18.1 (q)								
**6′**					60.9 (t)	60.9 (t)										

^b^ Interchangeable; * overlapped by the solvent signal.

**Table 3:** ^1^H NMR Spectroscopic Data for Compounds **5**–**8** in DMSO-*d*_6_.

No.	5a1–2	5b1–2	6a	6b	7a	7b	8a	8b
**5**			3.60 (1H, br s)	3.69 (1H, br s)	3.40 (1H, dd, 3.0, 0.9)	3.49 (1H, dd, 3.2, 1.0)	3.44 (1H, dd, 3.0, 0.8)	3.54 (1H, dd, 3.2, 0.9)
**7**	2.30 (3H, s)	2.33 (3H, s)	2.32 (3H, s)	2.34 (3H, s)	2.31 (3H, s)	2.34 (3H, s)	2.45 (3H, s)	2.48 (3H, s)
**8**	1.68 (1H, m)	1.68 (1H, m)	1.77 (1H, m)	1.77 (1H, m)	1.98 (1H, m)	1.98 (1H, m)	1.72 (1H, m)	1.72 (1H, m)
**9**	0.85/0.96, 1.15/1.74 (each 1H, m)	0.85/0.96, 1.15/1.74 (each 1H, m)	3.61 (1H, m)	3.61 (1H, m)	0.92 (3H, d, 7.0)	0.92 (3H, d, 7.0)	1.07, 1.22 (each 1H, m)	1.07, 1.22 (each 1H, m)
**10**	0.76/0.84 (3H, t, 7.1)	0.76/0.84 (3H, t, 7.1)	1.064 (3H, d, 6.2)	1.069 (3H, d, 6.2)			0.79 (3H, t, 7.4)	0.80 (3H, t, 7.4)
**11**	0.61/0.89 (3H, d, 6.9)	0.62/0.89 (3H, d, 6.9)	0.614 (3H, d, 6.8)	0.607 (3H, d, 6.8)	0.67 (3H, d, 7.0)	0.69 (3H, d, 7.0)	0.89 (3H, d, 7.0)	0.88 (3H, d, 6.9)
**1′**							3.33 (2H, m)	3.36 (2H, m)
**2′**							1.74 (2H, m)	1.74 (2H, m)
**3′**							2.12 (2H, t, 7.3)	2.12 (2H, t, 7.3)
**1-NH**	7.70/7.77 (1H, s)	7.47/7.55 (1H, s)	7.07 (1H, s)	6.84 (1H, s)	7.54 (1H, s)	7.32 (1H, s)	10.53 (1H, t, 5.9)	10.81 (1H, t, 5.9)
**12-NH**	8.59, 9.28 (each 1H, br s)	8.71, 9.47 (each 1H, br s)	8.70, 9.44 (each 1H, br s)	8.84, 9.67 (each 1H, br s)	8.66, 9.43 (each 1H, br s)	8.80, 9.67 (each 1H, br s)	6.82, 7.37 (each 1H, br s)	6.82, 7.37 (each 1H, br s)
**OH**	5.60/5.62 (1H, s)	5.63/5.65 (1H, s)						
**CONH**							7.62 (1H, br s)	7.35 (1H, br s)

## Data Availability

Data are contained within the article and Supplementary Materials.

## Supplementary Materials

The following supporting information can be downloaded at: https://www.mdpi.com/article/10.3390/jof10120809/s1, Figures S1–S56: The NMR, HRESIMS, and UV data for **1**–**8** are included.
