# Supplementary material for: New PKS/NRPS Tenuazamines A–H from the Endophytic Fungus Alternaria alternata FL7 Isolated from Huperzia serrata
Source: J Fungi (Basel). 2024 Nov 21;10(12):809. doi: 10.3390/jof10120809 (PMC11676839; doi:10.3390/jof10120809)

Supporting Information for

Novel PKS/NRPS Tenuazamines A–H from the Endophytic Fungus *Alternaria alternata* FL7 Isolated from *Huperzia serrata*

Hao Zhang[1], Zhibin Zhang[1], Yiwen Xiao[1, 2, *], Wen Wang[1], Boliang Gao[1], Yuhao Xie[1], Jiahao Xie[1], Xinhua Gao[1], Du Zhu[1, 2, *]

Abstract: In this paper, we present a novel class of hybrid polyketides, tenuazamines A–H (**1**–**8**), which exhibit a unique tautomeric equilibrium from *Alternaria alternata* FL7. The elucidation of the structures was achieved through a combination of NMR, HR-ESIMS, ECD diverse methods, with a focus on extensive spectroscopic data analysis. Notably, compounds **1**, **4**, **8**-**9** exhibited potent toxic effects on the growth of *Arabidopsis thaliana*. This research expands the structural diversity of tenuazonic acid compounds derived from endophytic fungi and provides potential hit compounds for the development of herbicides.

Keywords: *Alternaria alternata*; *Huperzia serrata*; tenuazamine; tautomer; herbicides.

CONTENT

Fig. S1. The chemical structure of TeAs reported........1
Fig. S2. ^{1}H NMR spectrum (DMSO-d_6, 600Hz) of 1........1
Fig. S3. ^{13}C NMR spectrum (DMSO-d_6, 150Hz) of 1........2
Fig. S4. ^{1}H-^{1}H COSY spectrum (DMSO-d_6, 600Hz) of 1........2
Fig. S5. HMBC spectrum (DMSO-d_6, 600Hz) of 1........3
Fig. S6. HSQC spectrum (DMSO-d_6, 600Hz) of 1........3
Fig. S7. UV spectrum (MeOH) of 1........4
Fig. S8. (+)-HR-ESI-MS of compound 1........4
Fig. S9. ^{1}H NMR spectrum (DMSO-d_6, 400Hz) of 2........4
Fig. S10. ^{13}C NMR spectrum (DMSO-d_6, 100Hz) of 2........5
Fig. S11. ^{1}H-^{1}H COSY spectrum (DMSO-d_6, 400Hz) of 2........5
Fig. S12. HMBC spectrum (DMSO-d_6, 400Hz) of 2........6
Fig. S13. HSQC spectrum (DMSO-d_6, 400Hz) of 2........6
Fig. S14. UV spectrum (MeOH) of 2........7
Fig. S15. (+)-HR-ESI-MS of compound 2........7
Fig. S16. ^{1}H NMR spectrum (DMSO-d_6, 400Hz) of 3........7
Fig. S17. ^{13}C NMR spectrum (DMSO-d_6, 400Hz) of 3........8
Fig. S18. ^{1}H-^{1}H COSY spectrum (DMSO-d_6, 400Hz) of 3........8
Fig. S19. HMBC spectrum (DMSO-d_6, 400Hz) of 3........9
Fig. S20. HSQC spectrum (DMSO-d_6, 400Hz) of 3........9
Fig. S21. UV spectrum (MeOH) of 3........10
Fig. S22. (+)-HR-ESI-MS of compound 3........10
Fig. S23. ^{1}H NMR spectrum (DMSO-d_6, 400Hz) of 4........10
Fig. S24. ^{13}C NMR spectrum (DMSO-d_6, 400Hz) of 4........11
Fig. S25. ^{1}H-^{1}H COSY spectrum (DMSO-d_6, 400Hz) of 4........11
Fig. S26. HMBC spectrum (DMSO-d_6, 400Hz) of 4........12
Fig. S27. HSQC spectrum (DMSO-d_6, 400Hz) of 4........12
Fig. S28. UV spectrum (MeOH) of 4........13
Fig. S29. (+)-HR-ESI-MS of compound 4........13
Fig. S30. ^{1}H NMR spectrum (DMSO-d_6, 400Hz) of 5........13
Fig. S31. ^{13}C NMR spectrum (DMSO-d_6, 400Hz) of 5........14
Fig. S32. ^{1}H-^{1}H COSY spectrum (DMSO-d_6, 400Hz) of 5........14
Fig. S33. HMBC spectrum (DMSO-d_6, 400Hz) of 5........15
Fig. S34. HSQC spectrum (DMSO-d_6, 400Hz) of 5........15
Fig. S35. UV spectrum (MeOH) of 5........16
Fig. S36. (+)-HR-ESI-MS of compound 5........16
Fig. S37. ^{1}H NMR spectrum (DMSO-d_6, 400Hz) of 6........16
Fig. S38. ^{13}C NMR spectrum (DMSO-d_6, 400Hz) of 6........17
Fig. S39. ^{1}H-^{1}H COSY spectrum (DMSO-d_6, 400Hz) of 6........17
Fig. S40. HMBC spectrum (DMSO-d_6, 400Hz) of 6........18
Fig. S41. HSQC spectrum (DMSO-d_6, 400Hz) of 6........18
Fig. S42. UV spectrum (MeOH) of 6........19

Fig. S43. (+)-HR-ESI-MS of compound 6..19
Fig. S44. ^{1}H NMR spectrum (DMSO-*d_6*, 400Hz) of 7...19
Fig. S45. ^{13}C NMR spectrum (DMSO-*d_6*, 400Hz) of 7..20
Fig. S46. ^{1}H-^{1}H COSY spectrum (DMSO-*d_6*, 400Hz) of 7...20
Fig. S47. HMBC spectrum (DMSO-*d_6*, 400Hz) of 7...21
Fig. S48. HSQC spectrum (DMSO-*d_6*, 400Hz) of 7..21
Fig. S49. UV spectrum (MeOH) of 7..22
Fig. S50. (+)-HR-ESI-MS of compound 7..22
Fig. S51. ^{1}H NMR spectrum (DMSO-*d_6*, 600Hz) of 8...22
Fig. S52. ^{13}C NMR spectrum (DMSO-*d_6*, 600Hz) of 8..23
Fig. S53. ^{1}H-^{1}H COSY spectrum (DMSO-*d_6*, 600Hz) of 8...23
Fig. S54. HMBC spectrum (DMSO-*d_6*, 600Hz) of 8...24
Fig. S55. HSQC spectrum (DMSO-*d_6*, 600Hz) of 8..24
Fig. S56. (+)-HR-ESI-MS of compound 8..25

Fig. S1. The chemical structure of TeAs that reported

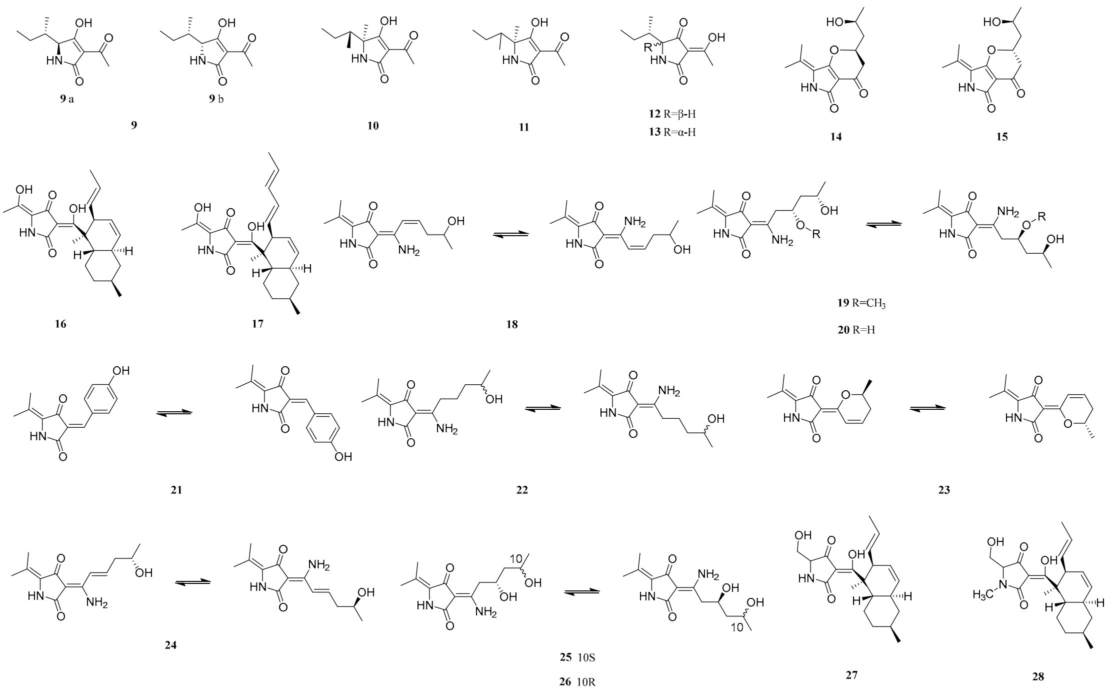

Fig. S2. ^{1}H NMR spectrum (DMSO-*d6*, 600Hz) of 1

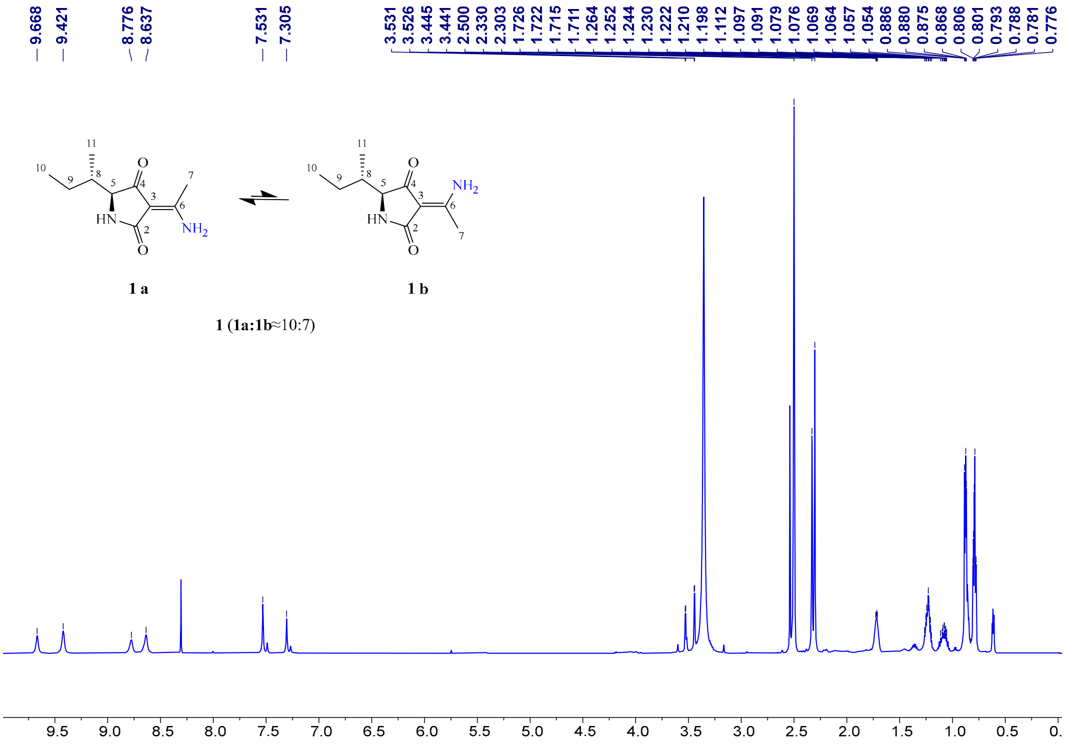

Fig. S3. ^{13}C NMR spectrum (DMSO-*d6*, 150Hz) of 1

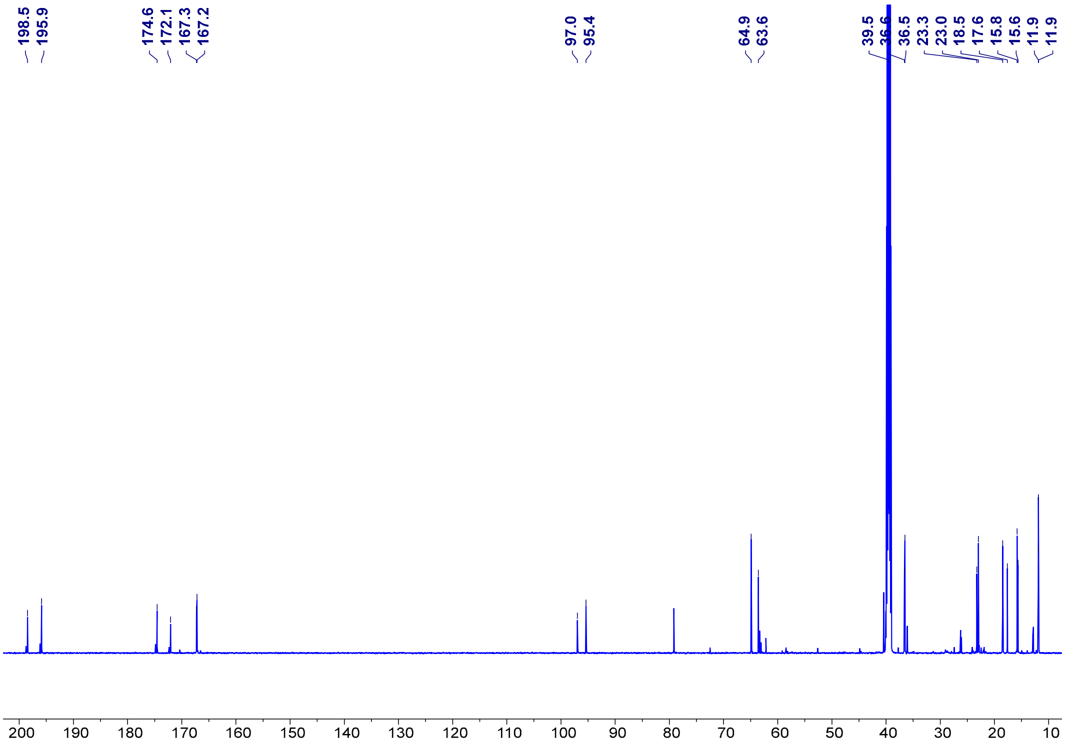

Fig. S4. ^{1}H-^{1}H COSY spectrum (DMSO-*d6*, 600Hz) of 1

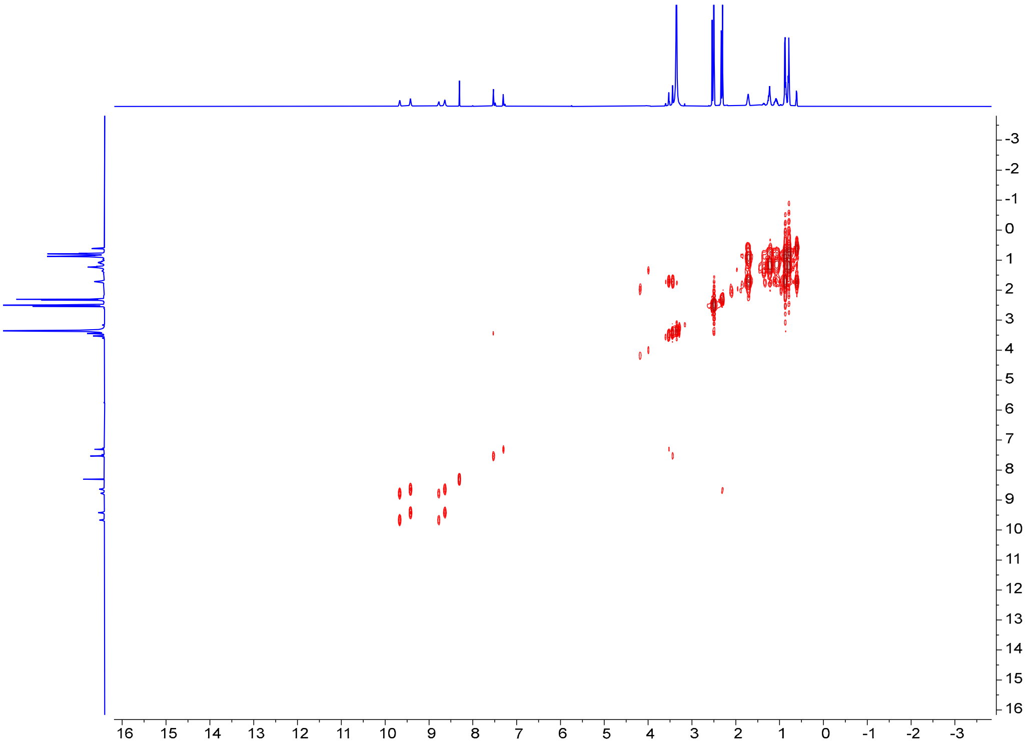

Fig. S5. HMBC spectrum (DMSO-*d6*, 600Hz) of 1

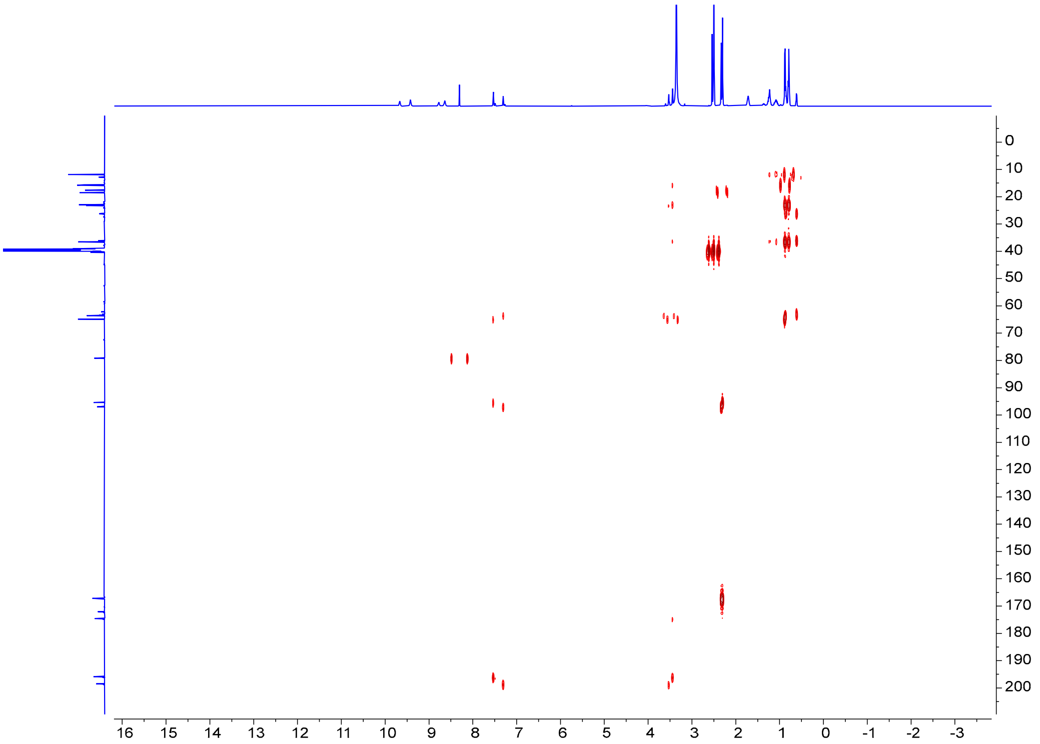

Fig. S6. HSQC spectrum (DMSO-*d6*, 600Hz) of 1

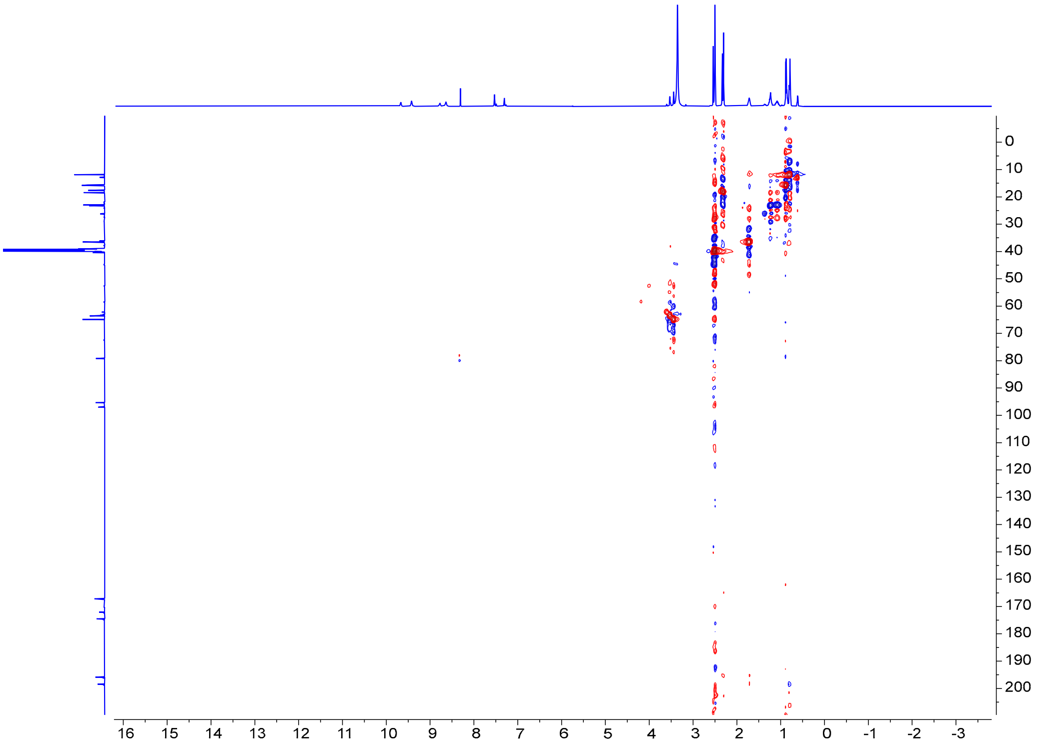

Fig. S7. UV spectrum (MeOH) of 1

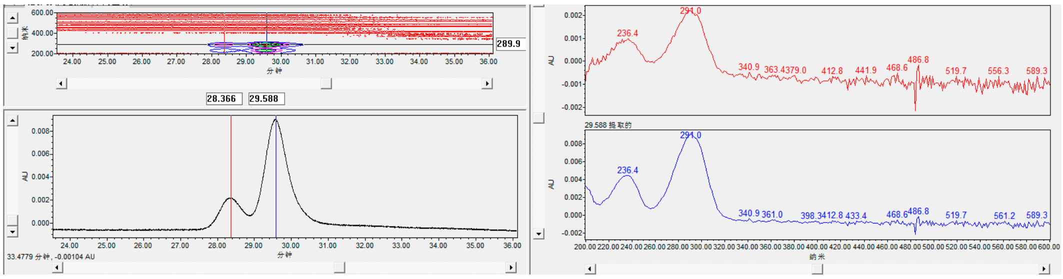

Fig. S8. (+)-HR-ESI-MS of compound 1

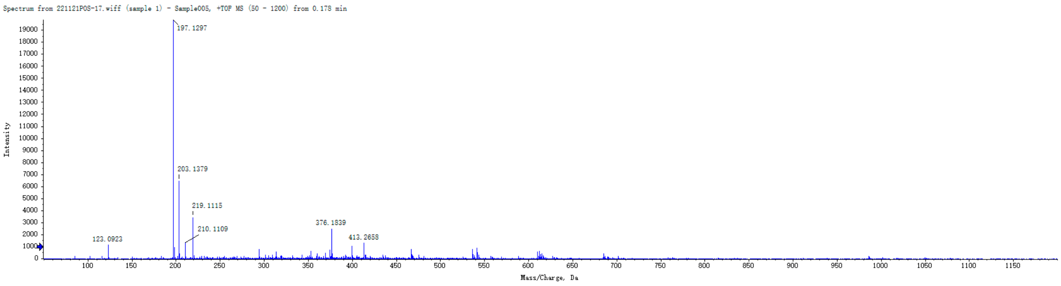

Fig. S9. ¹H NMR spectrum (DMSO-*d6*, 400Hz) of 2

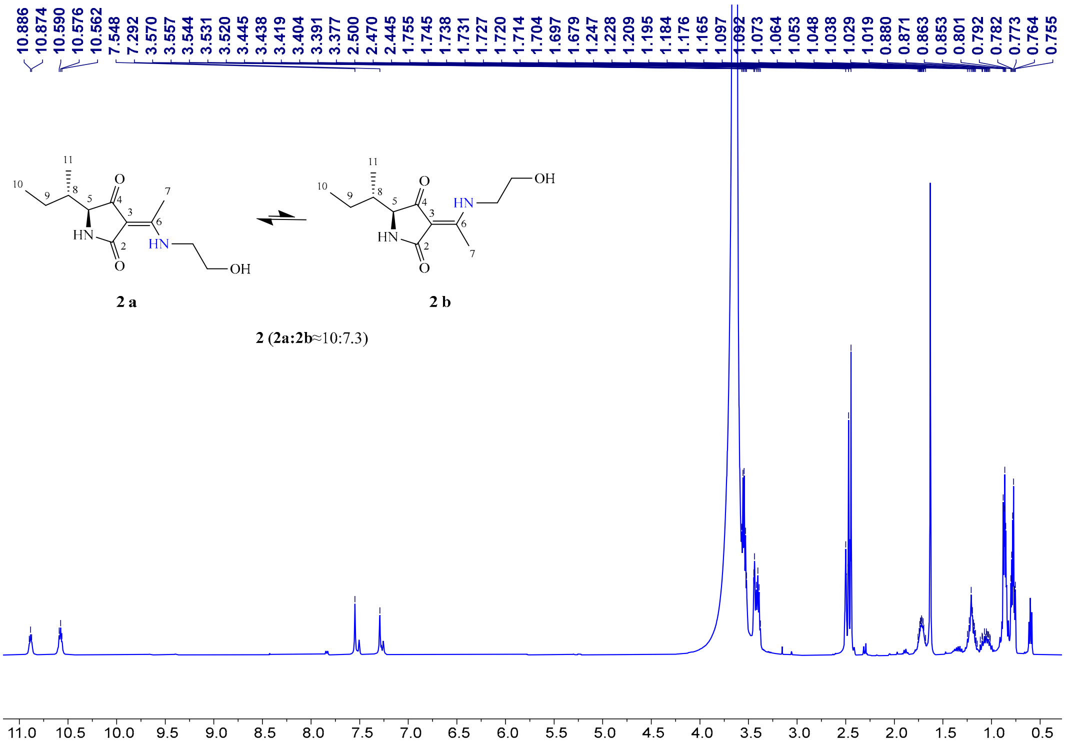

Fig. S10. ^{13}C NMR spectrum (DMSO-*d6*, 100Hz) of 2

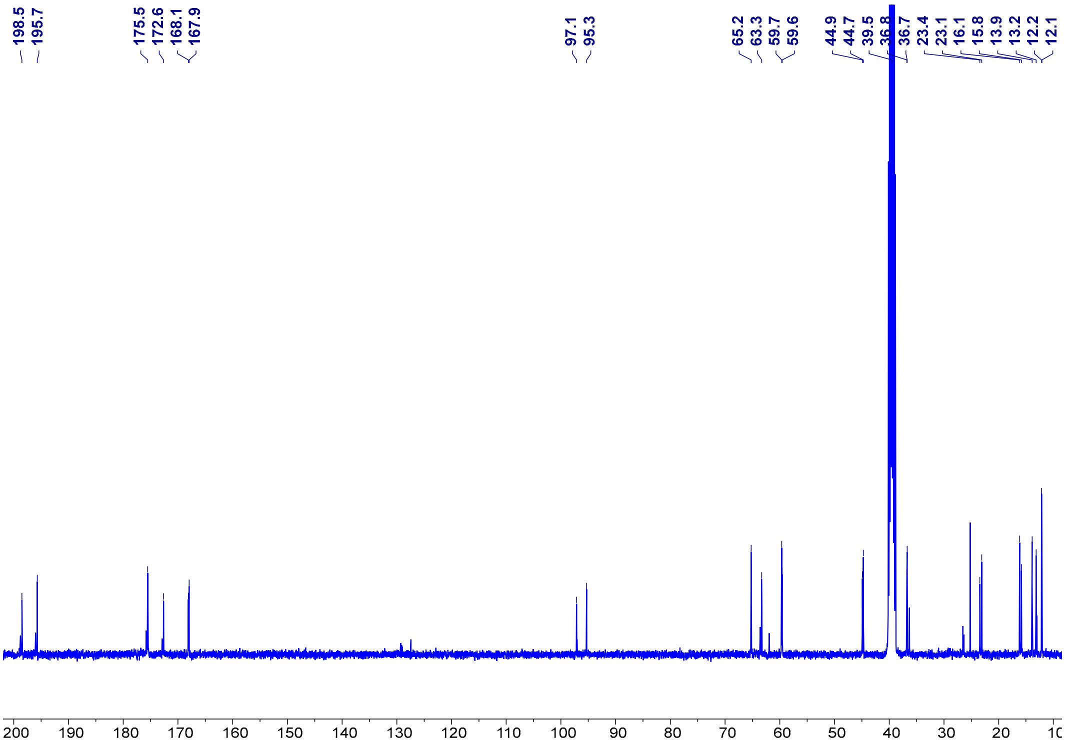

Fig. S11. ^{1}H-^{1}H COSY spectrum (DMSO-*d6*, 400Hz) of 2

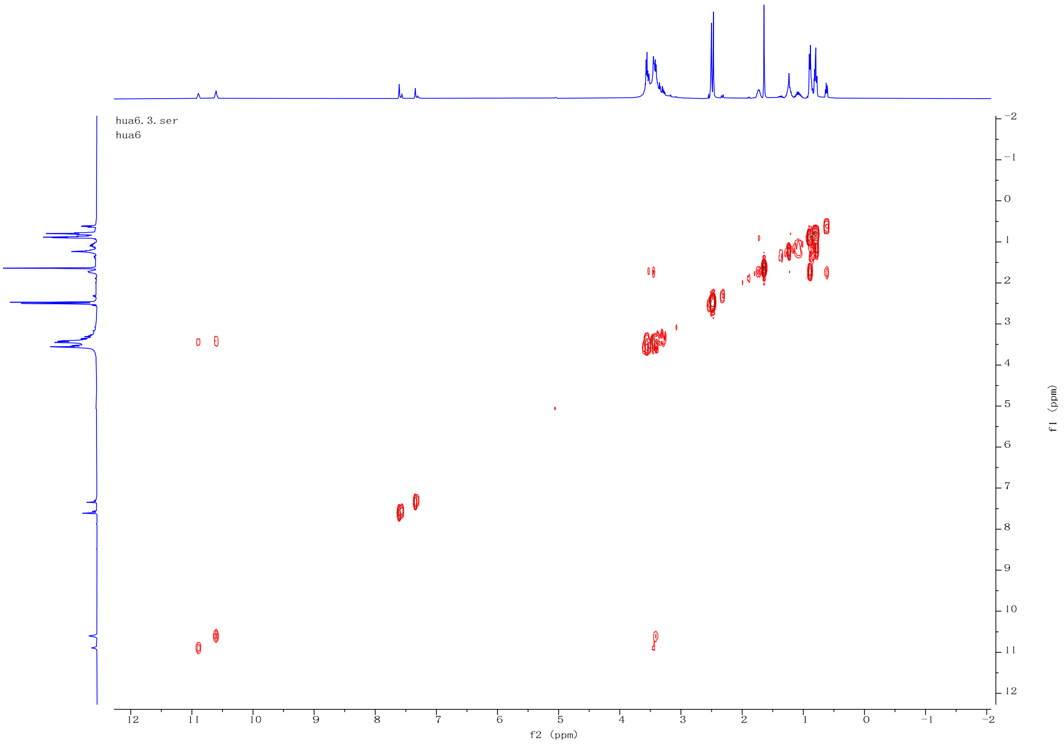

Fig. S12. HMBC spectrum (DMSO-*d6*, 400Hz) of 2

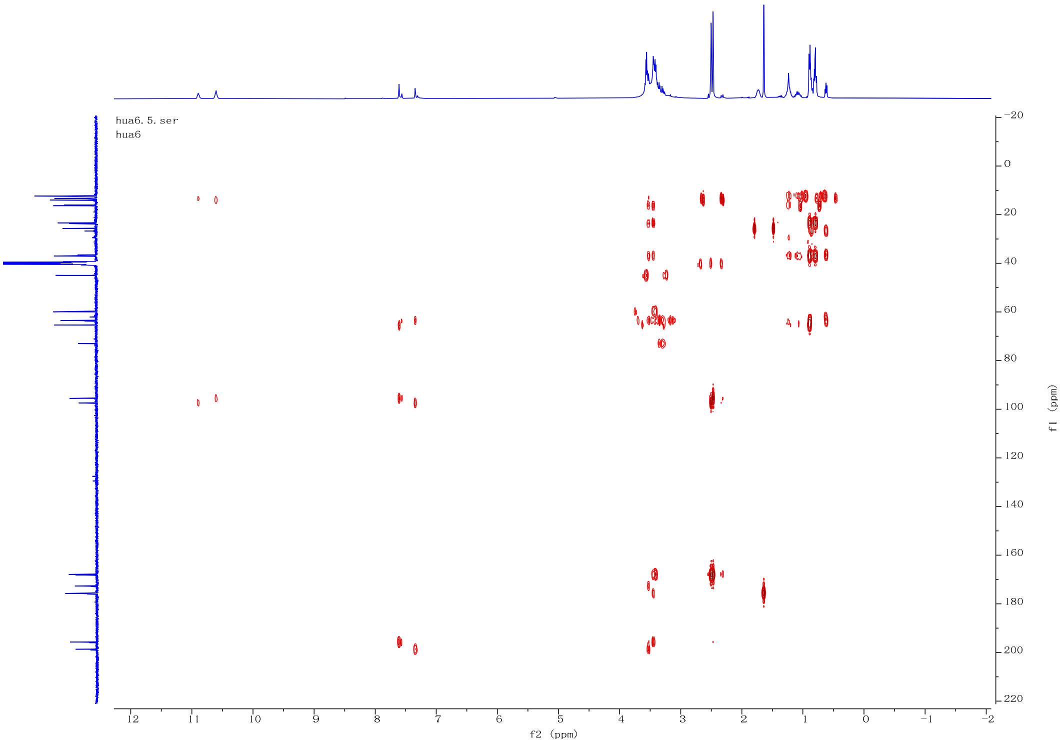

Fig. S13. HSQC spectrum (DMSO-*d6*, 400Hz) of 2

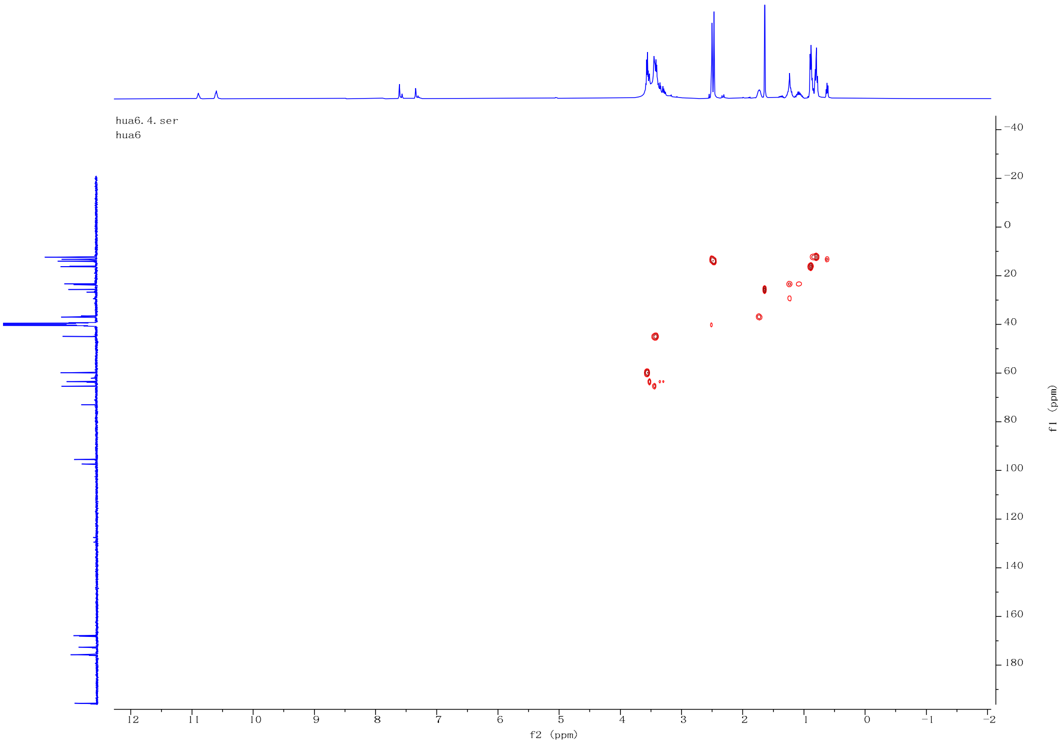

Fig. S14. UV spectrum (MeOH) of 2

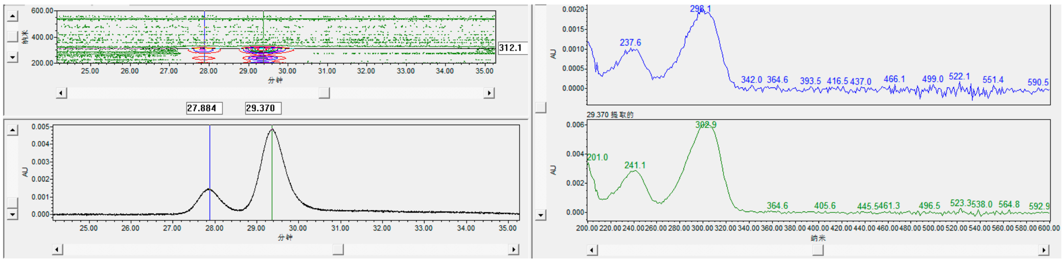

Fig. S15. (+)-HR-ESI-MS of compound 2

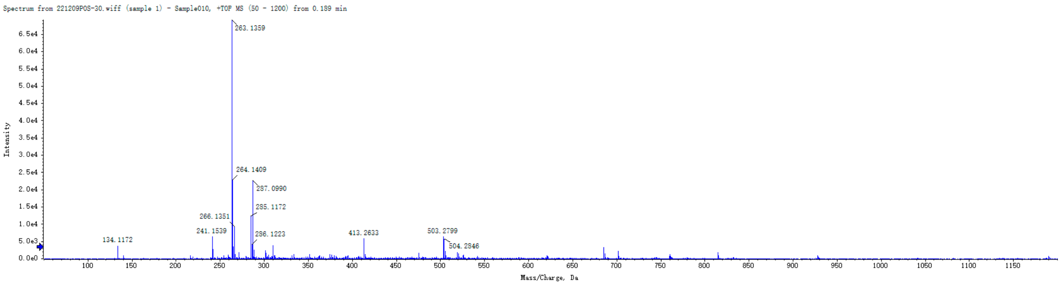

Fig. S16. ^{1}H NMR spectrum (DMSO-*d6*, 400Hz) of 3

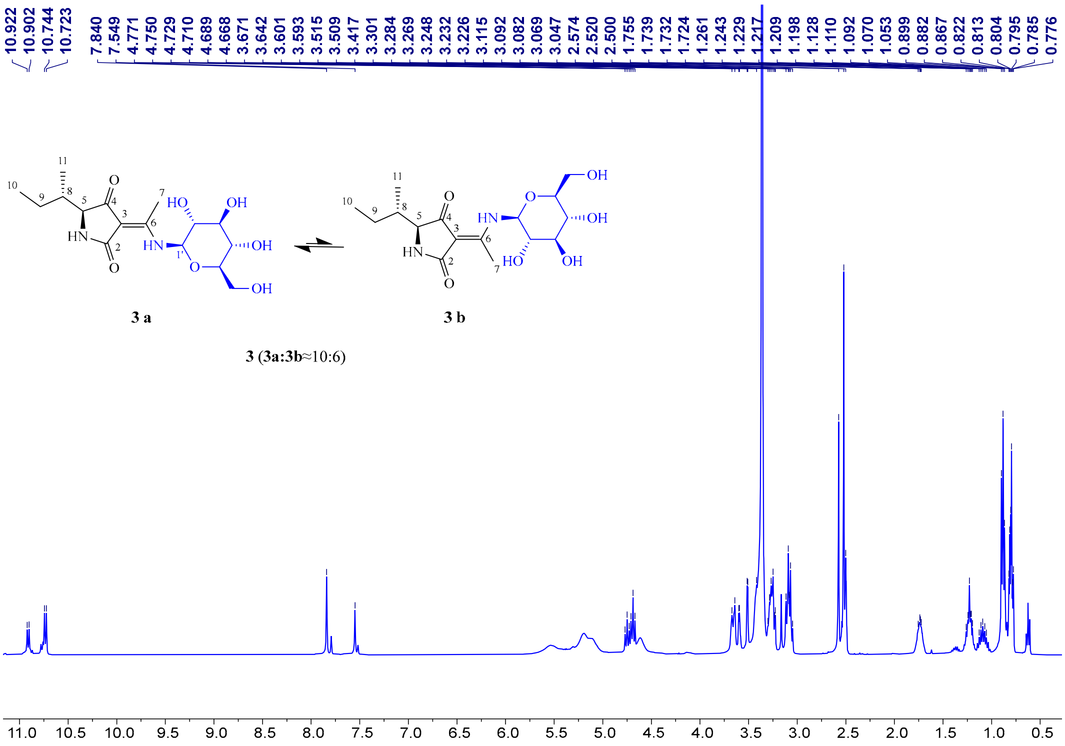

Fig. S17. ^{13}C NMR spectrum (DMSO-*d6*, 400Hz) of 3

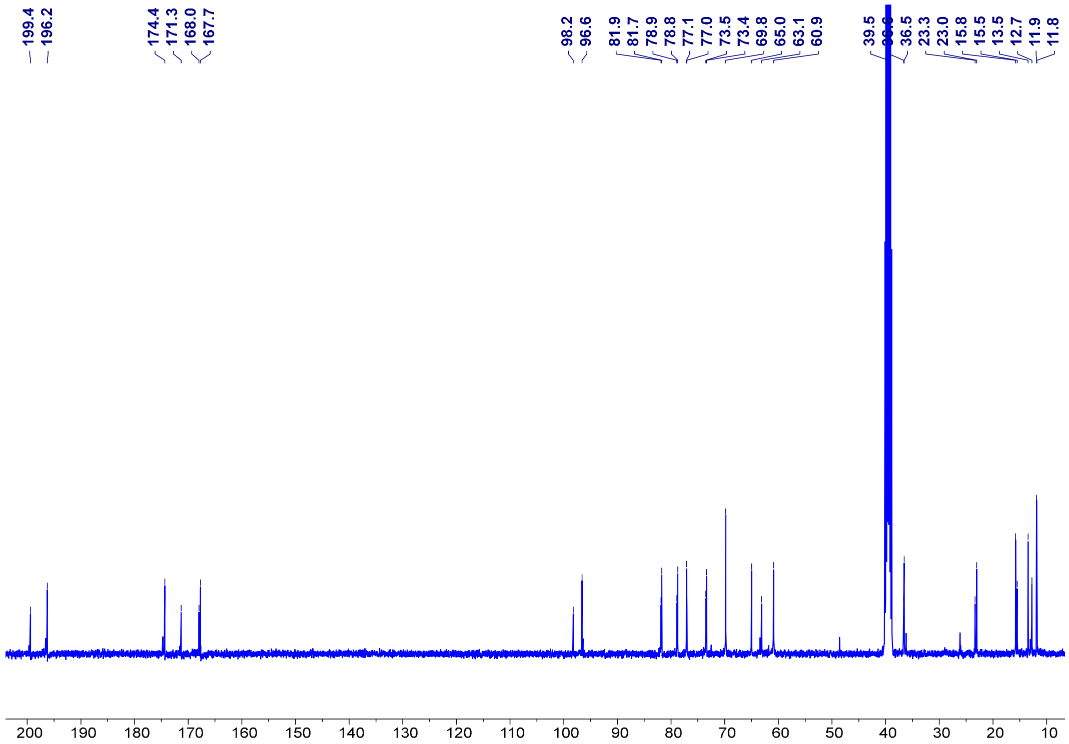

Fig. S18. ^{1}H-^{1}H COSY spectrum (DMSO-*d6*, 400Hz) of 3

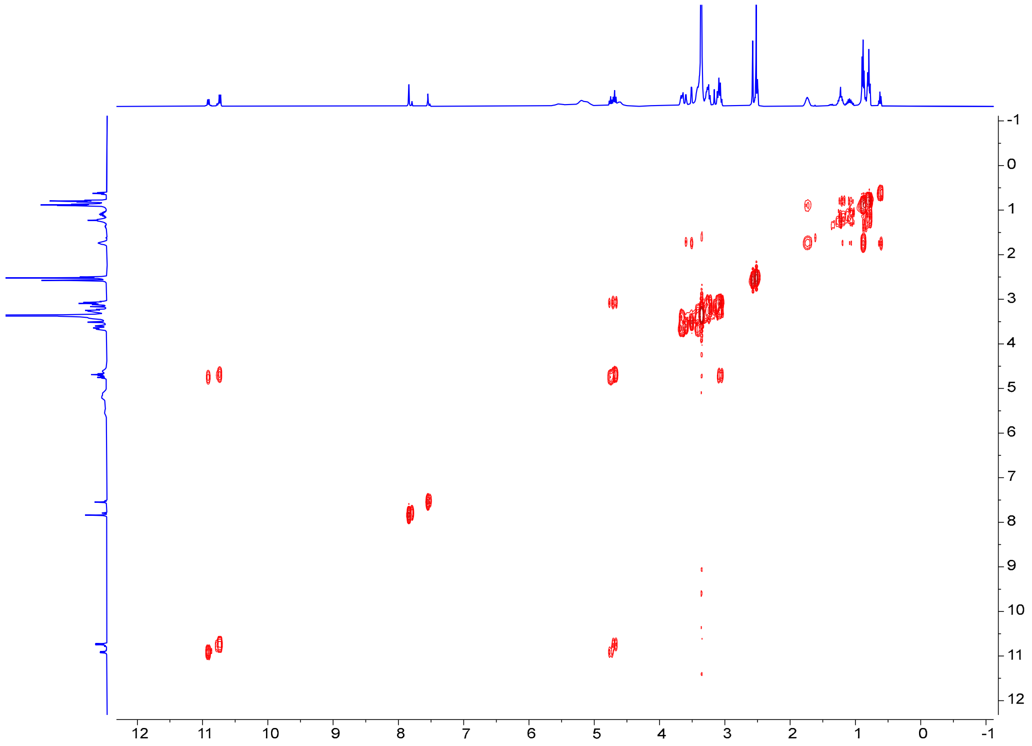

Fig. S19. HMBC spectrum (DMSO-*d6*, 400Hz) of 3

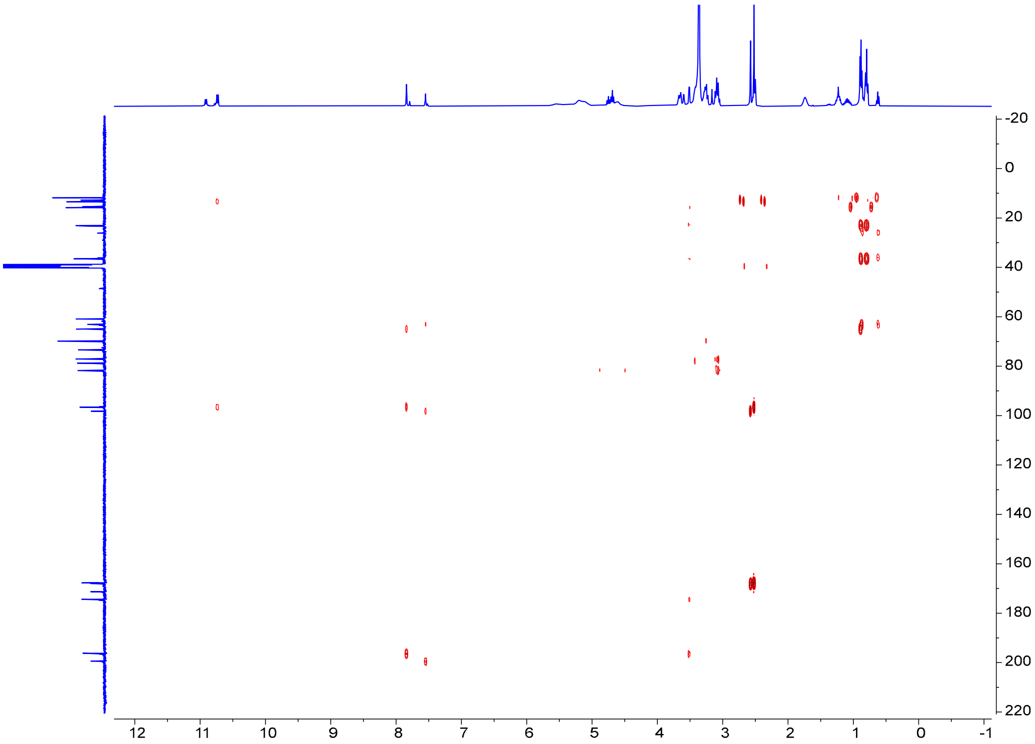

Fig. S20. HSQC spectrum (DMSO-*d6*, 400Hz) of 3

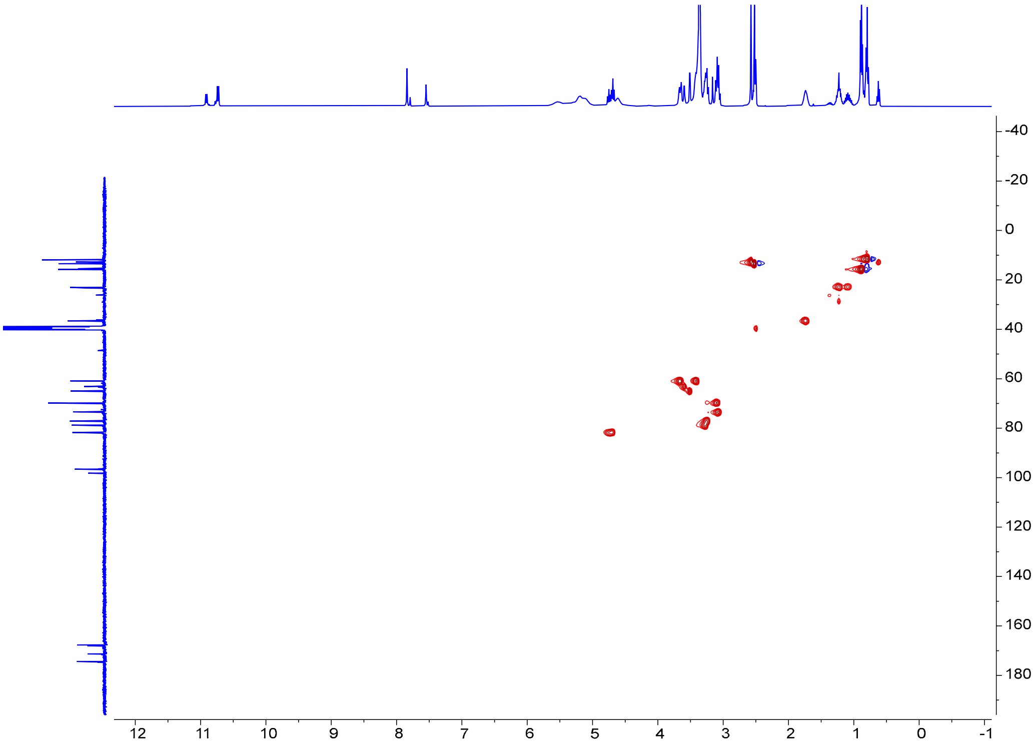

Fig. S21. UV spectrum (MeOH) of 3

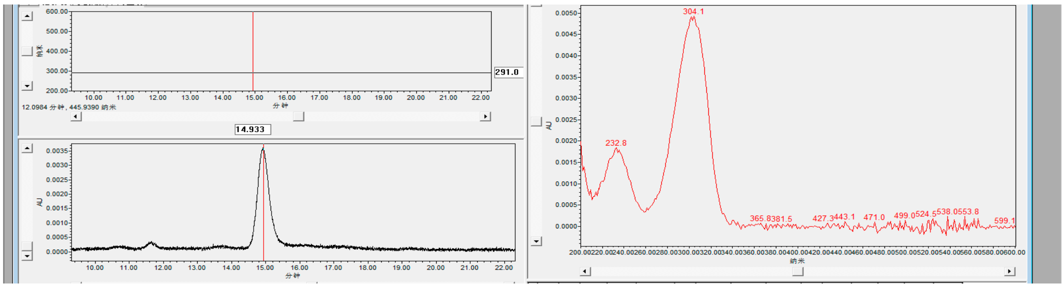

Fig. S22. (+)-HR-ESI-MS of compound 3

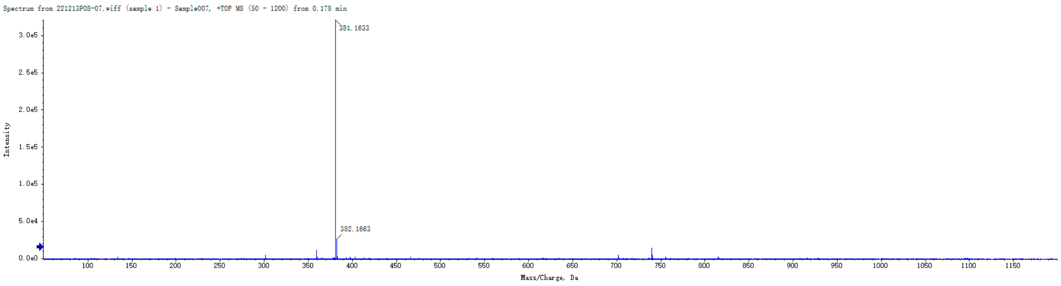

Fig. S23. ^{1}H NMR spectrum (DMSO-*d6*, 400Hz) of 4

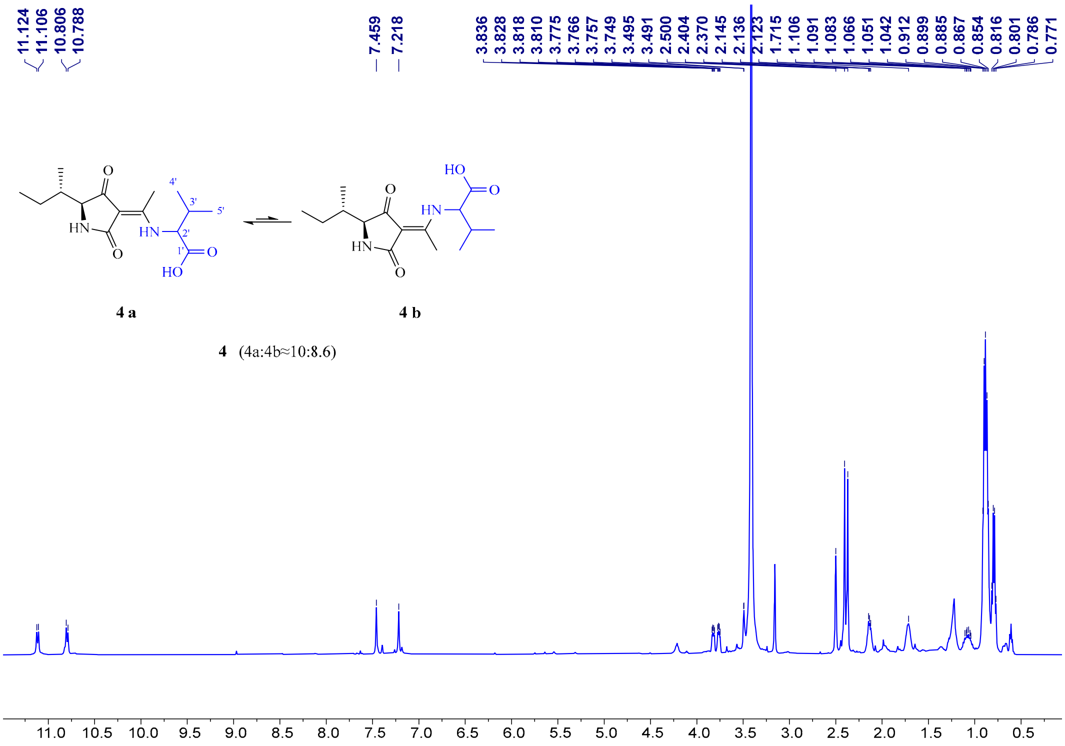

Fig. S24. ^{13}C NMR spectrum (DMSO-*d6*, 400Hz) of 4

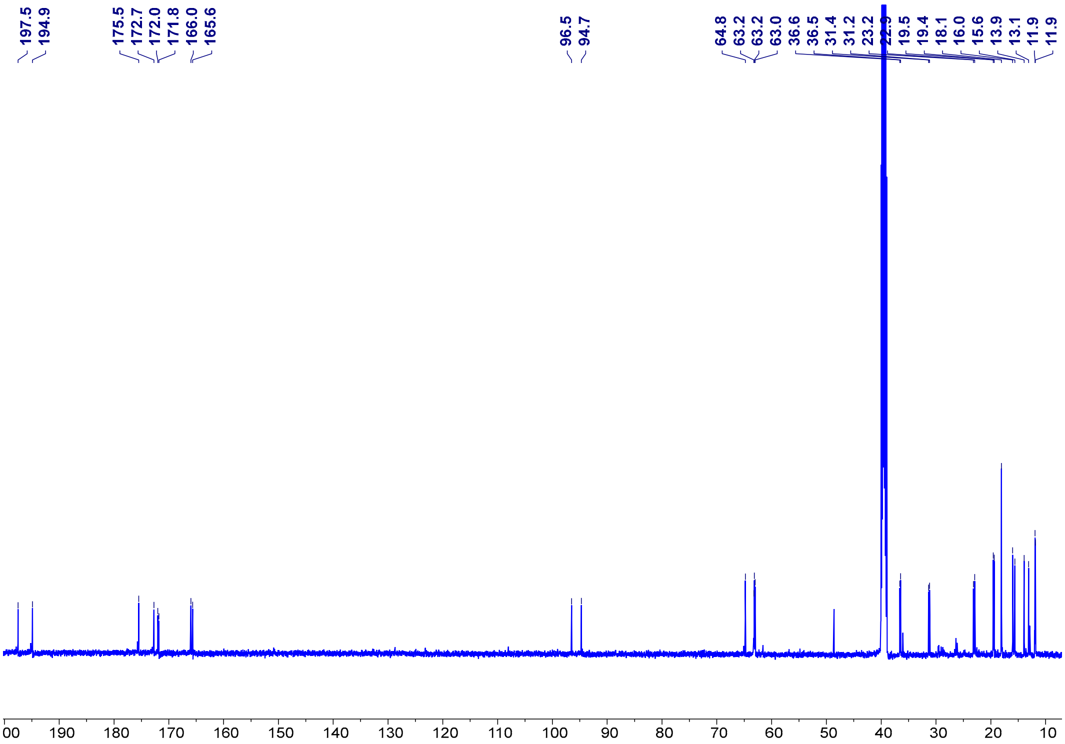

Fig. S25. ^{1}H-^{1}H COSY spectrum (DMSO-*d6*, 400Hz) of 4

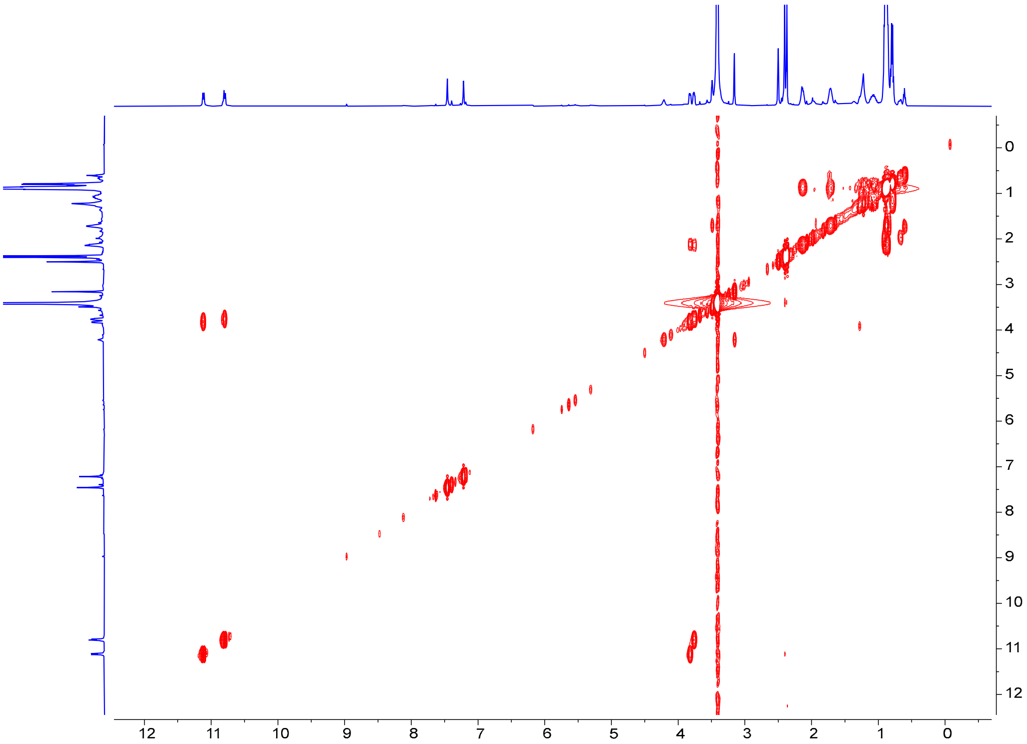

Fig. S26. HMBC spectrum (DMSO-*d6*, 400Hz) of 4

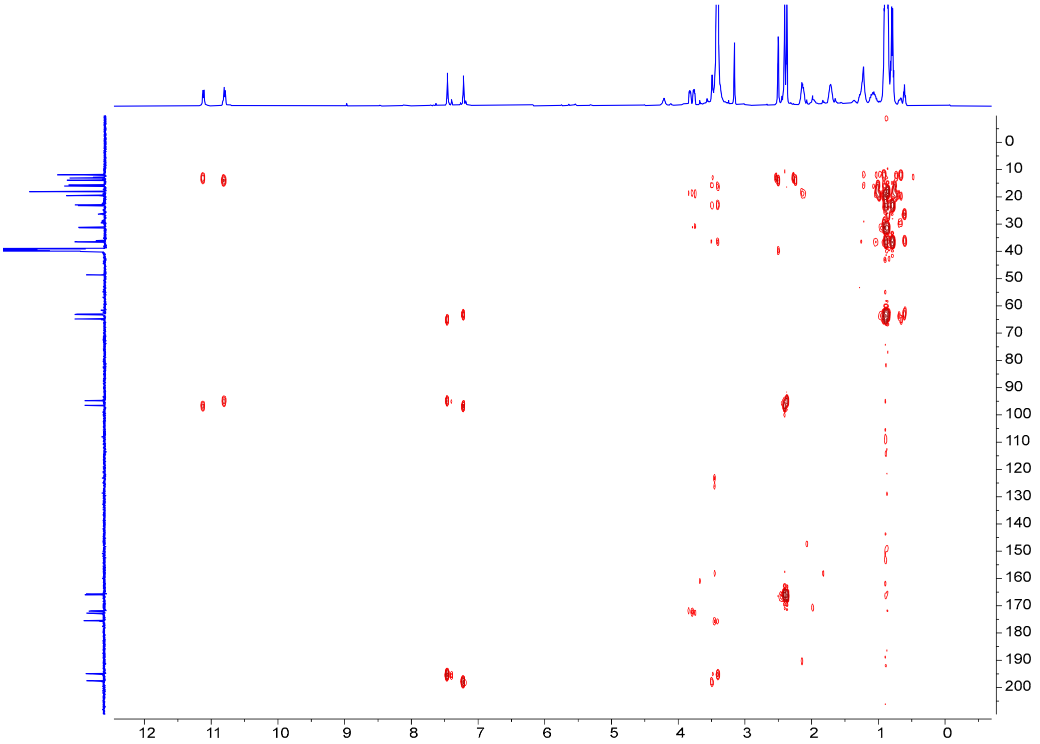

Fig. S27. HSQC spectrum (DMSO-*d6*, 400Hz) of 4

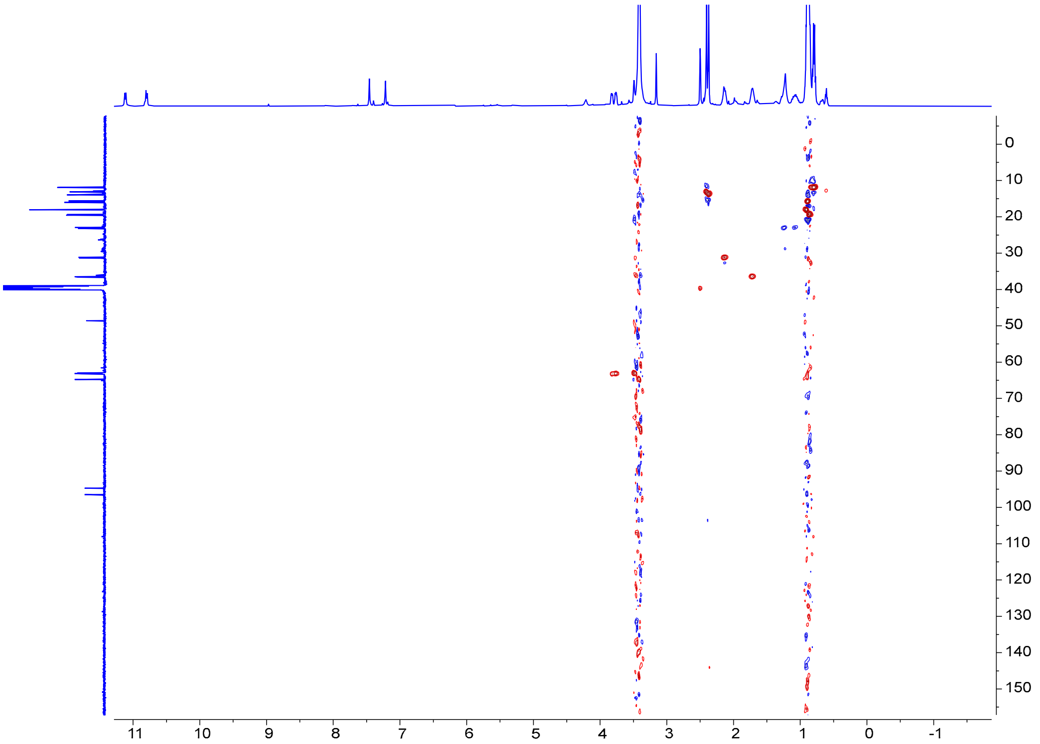

Fig. S28. UV spectrum (MeOH) of 4

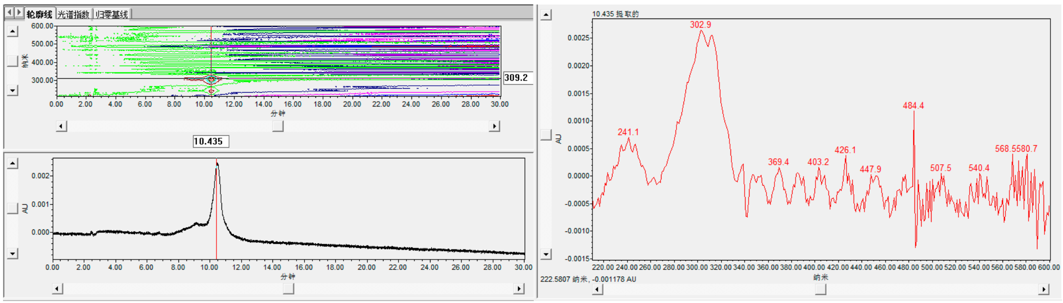

Fig. S29. (+)-HR-ESI-MS of compound 4

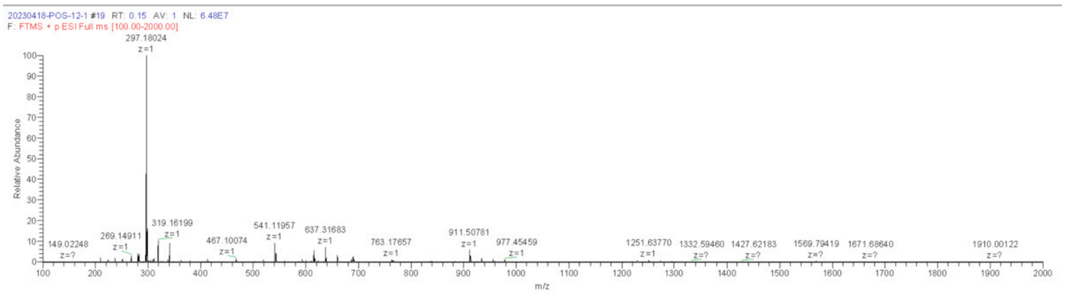

Fig. S30. ^{1}H NMR spectrum (DMSO-*d6*, 400Hz) of 5

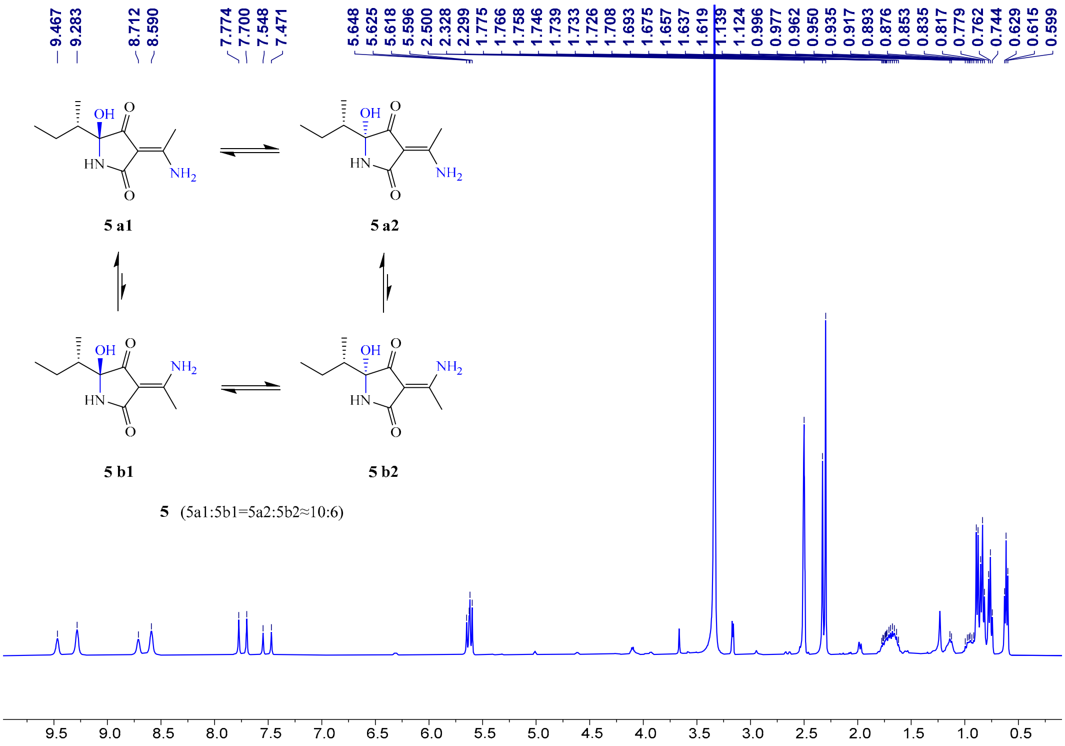

Fig. S31. ^{13}C NMR spectrum (DMSO-*d6*, 400Hz) of 5

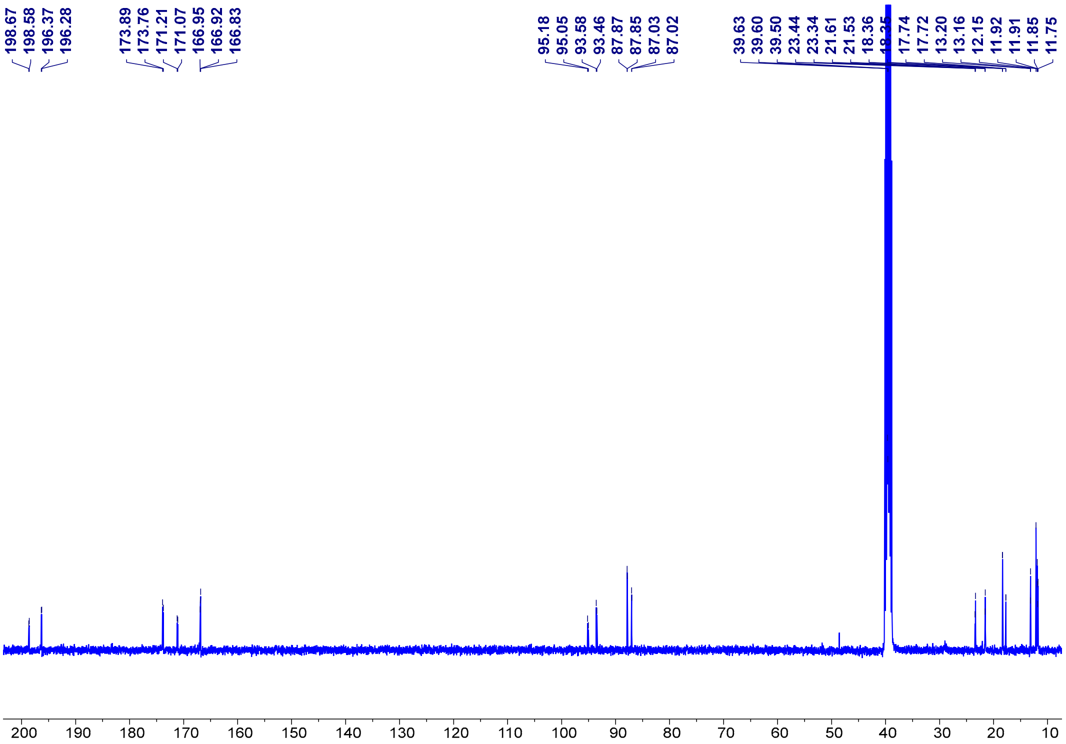

Fig. S32. ^{1}H-^{1}H COSY spectrum (DMSO-*d6*, 400Hz) of 5

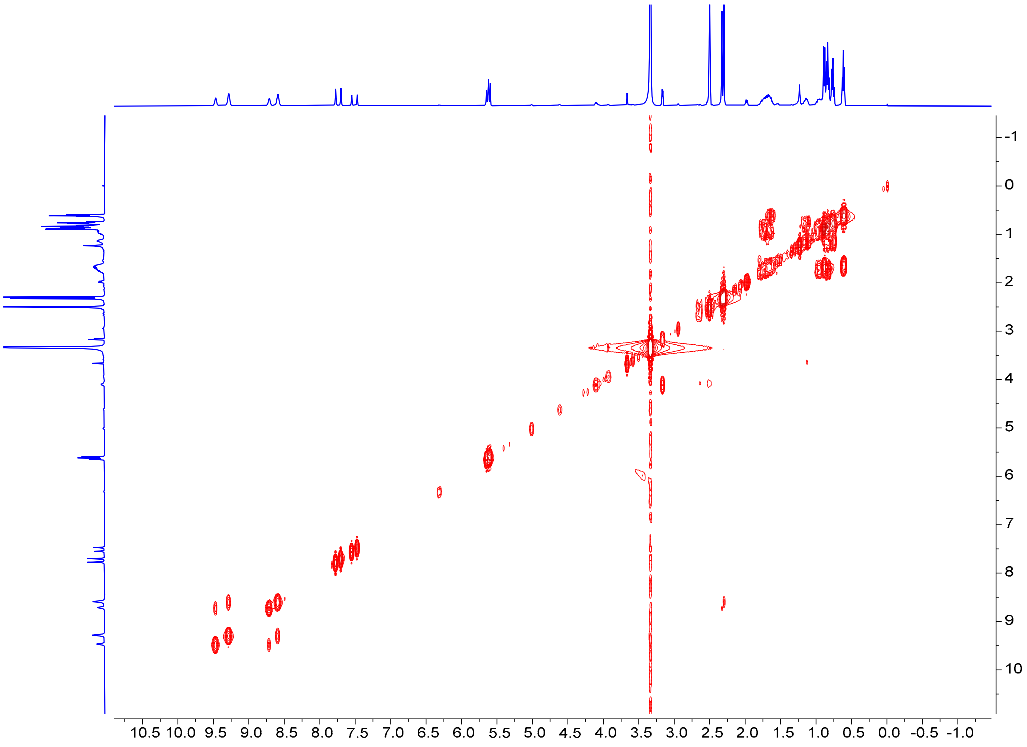

Fig. S33. HMBC spectrum (DMSO-*d6*, 400Hz) of 5

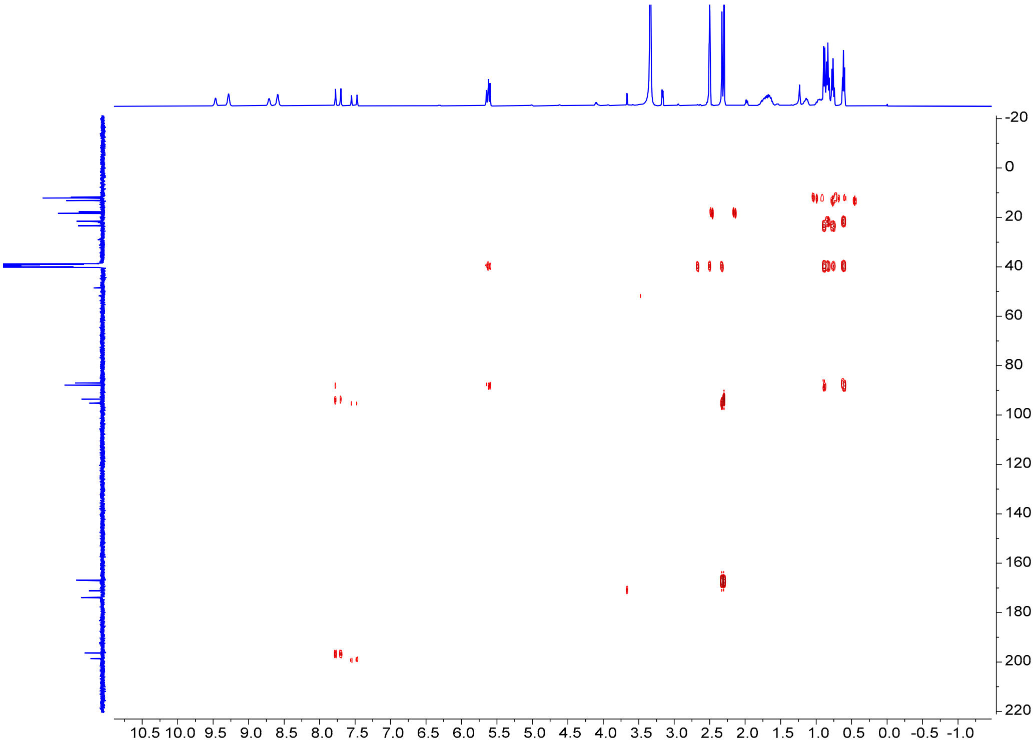

Fig. S34. HSQC spectrum (DMSO-*d6*, 400Hz) of 5

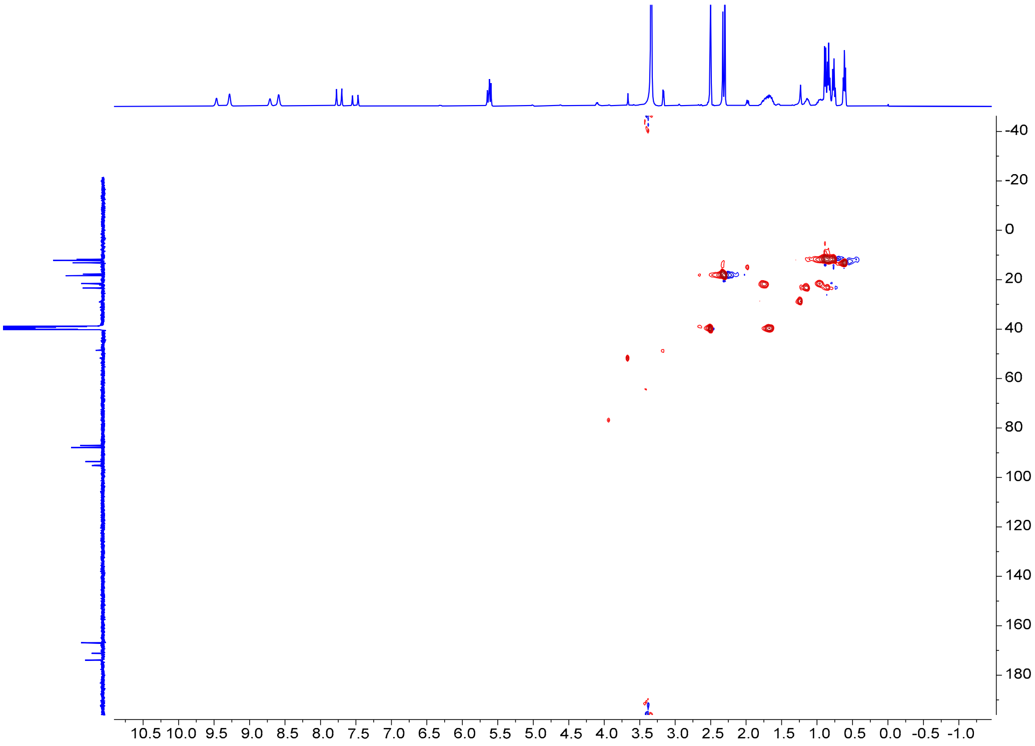

Fig. S35. UV spectrum (MeOH) of 5

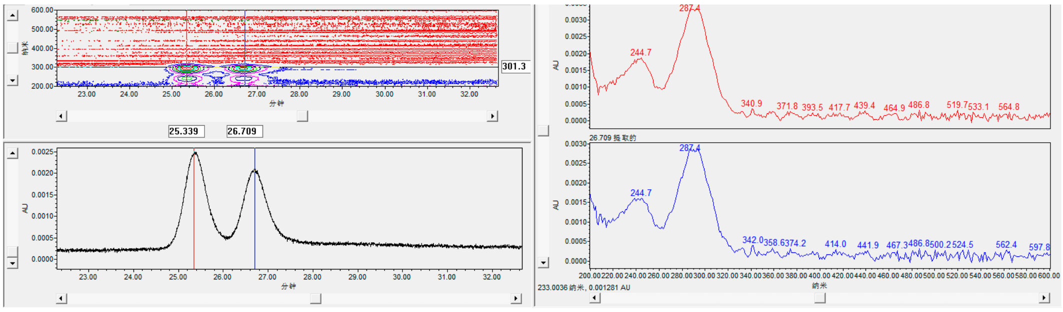

Fig. S36. (-)-HR-ESI-MS of compound 5

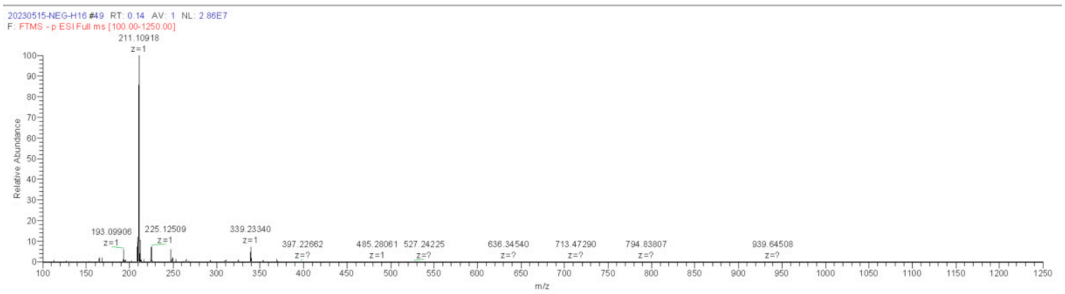

Fig. S37. ¹H NMR spectrum (DMSO-*d6*, 400Hz) of 6

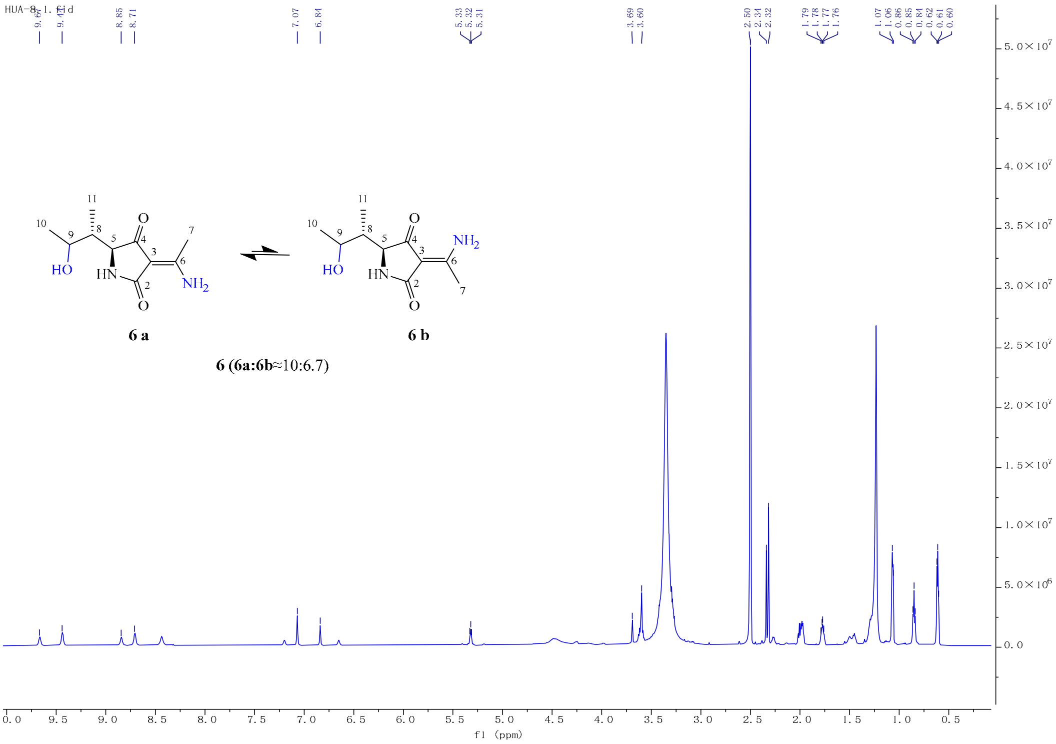

Fig. S38. ^{13}C **NMR spectrum (DMSO-*d6*, 400Hz) of 6**

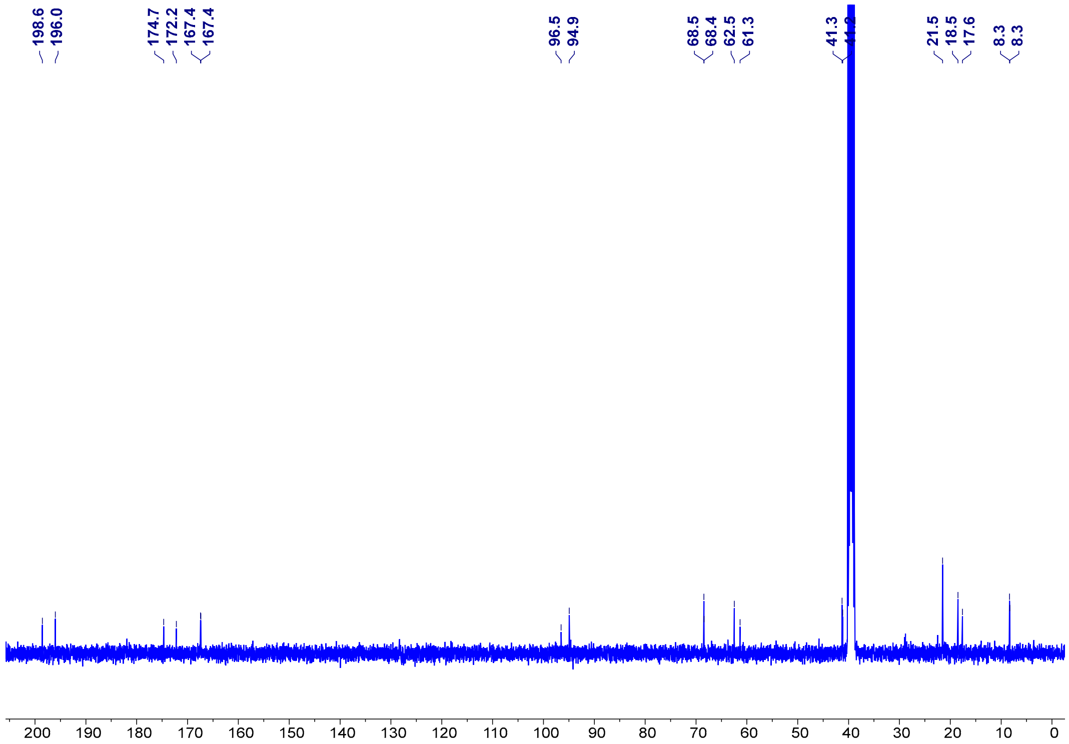

Fig. S39. ^{1}H-^{1}H **COSY spectrum (DMSO-*d6*, 400Hz) of 6**

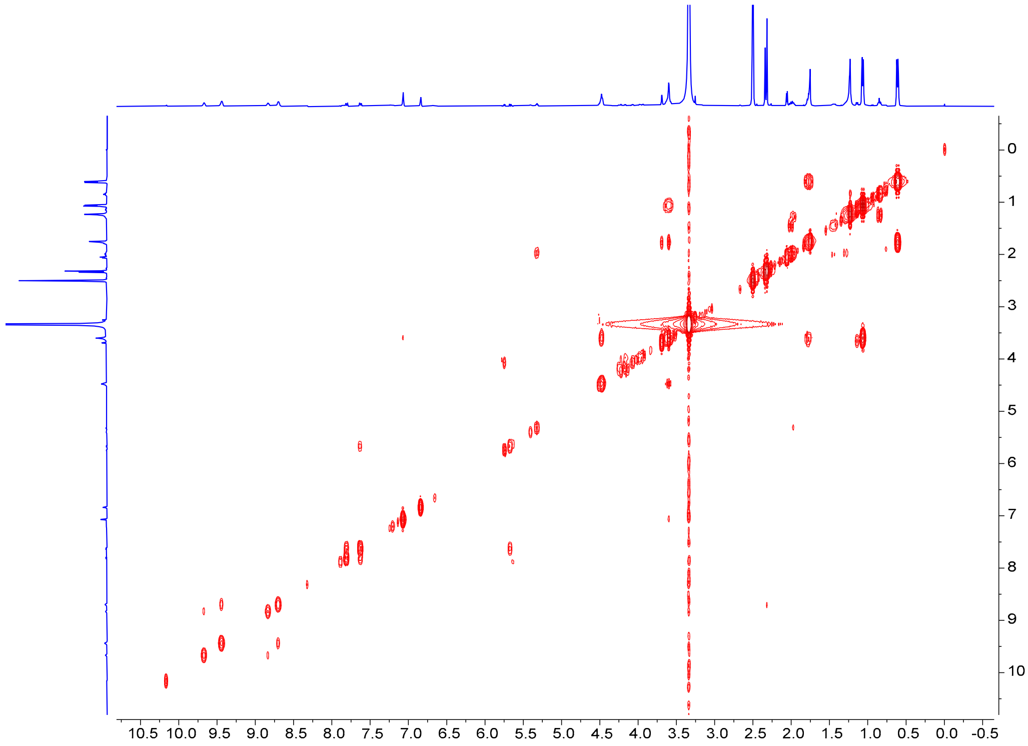

Fig. S40. HMBC spectrum (DMSO-*d6*, 400Hz) of 6

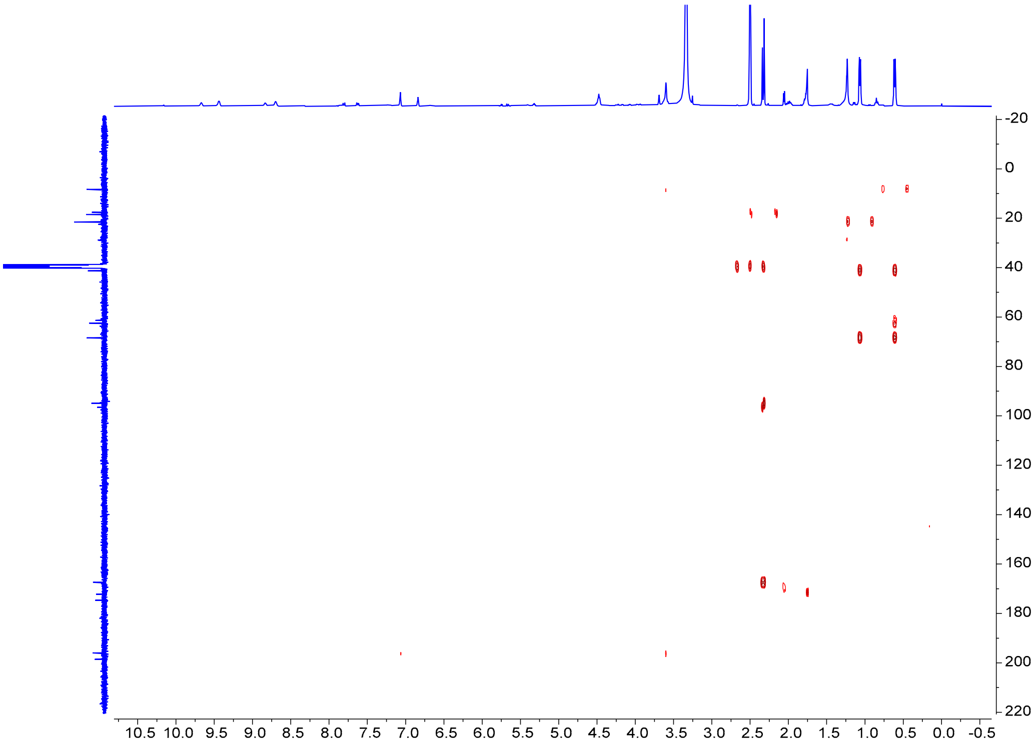

Fig. S41. HSQC spectrum (DMSO-*d6*, 400Hz) of 6

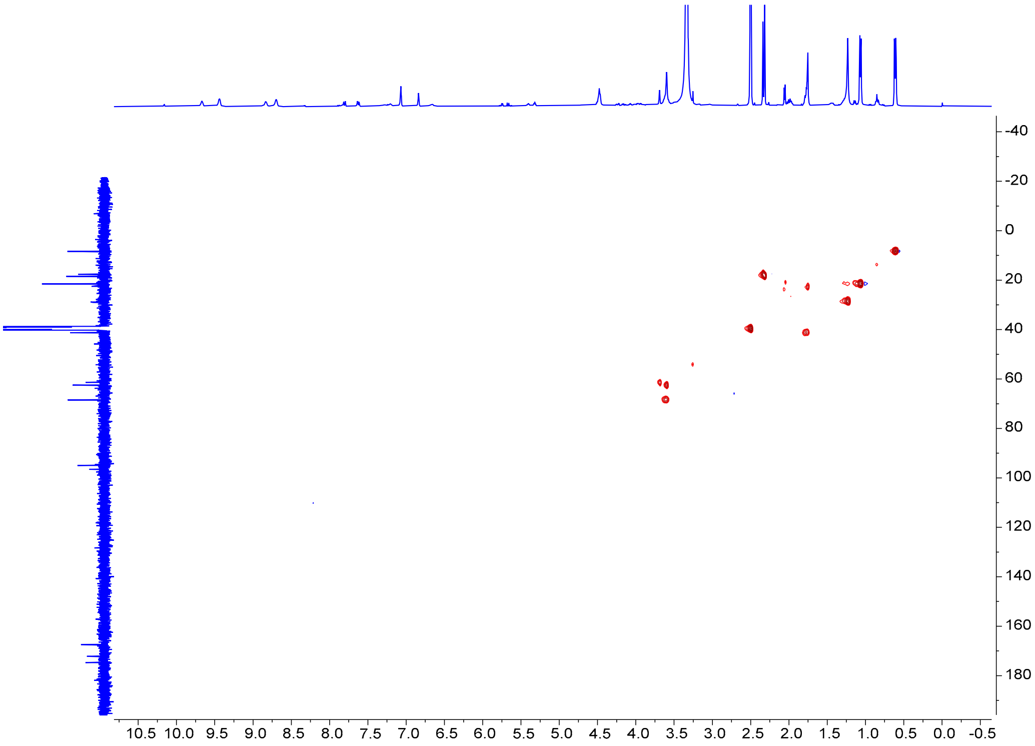

Fig. S42. UV spectrum (MeOH) of 6

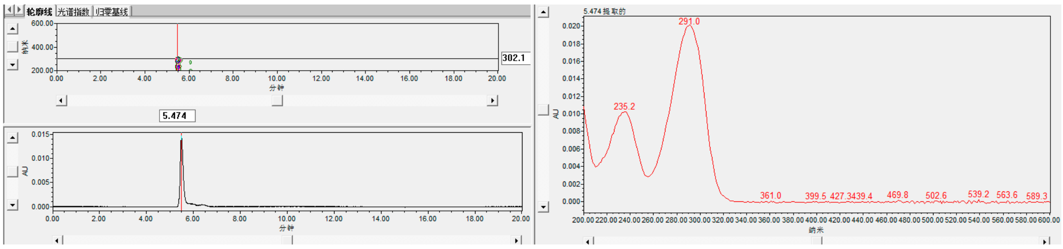

Fig. S43. (+)-HR-ESI-MS of compound 6

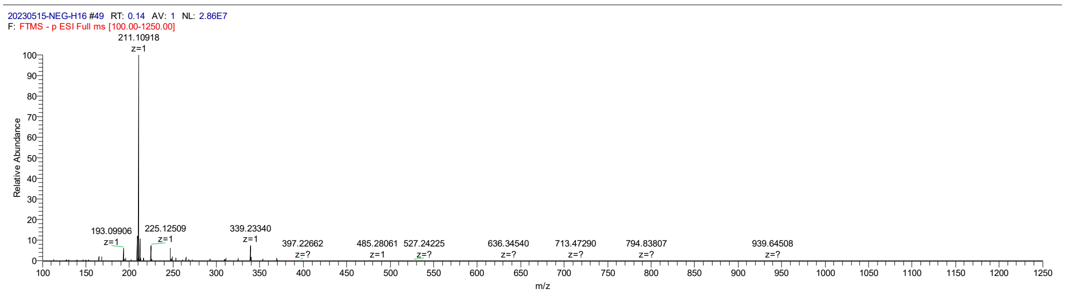

Fig. S44. ^{1}H NMR spectrum (DMSO-*d6*, 400Hz) of 7

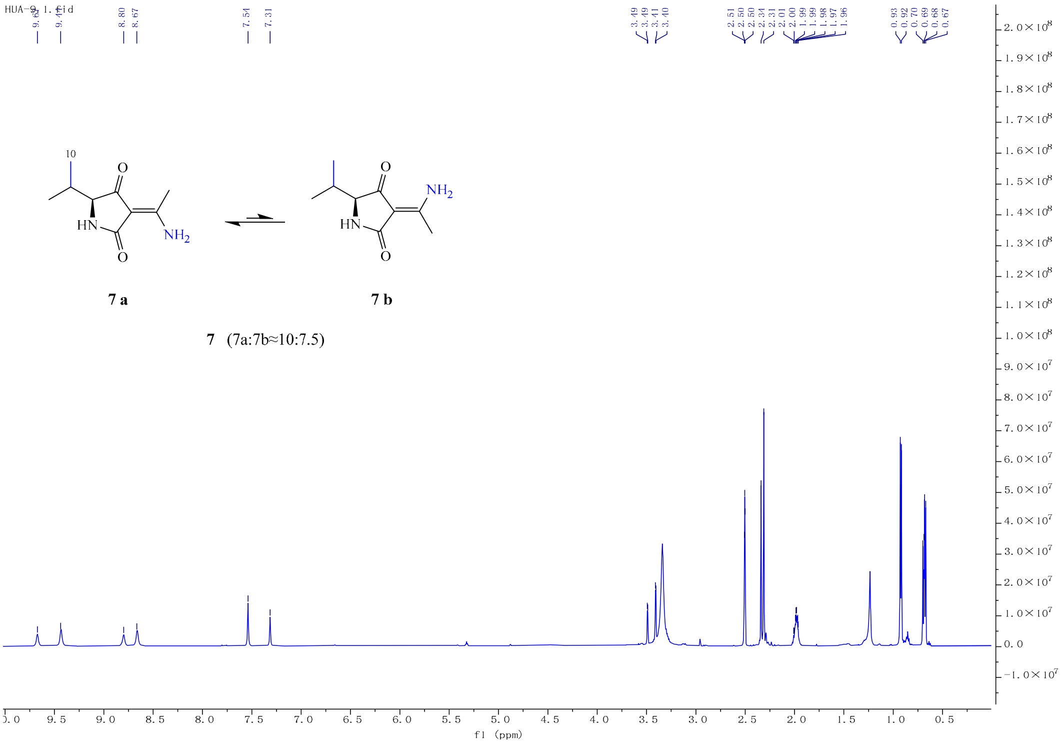

Fig. S45. ^{13}C **NMR spectrum (DMSO-*d6*, 400Hz) of 7**

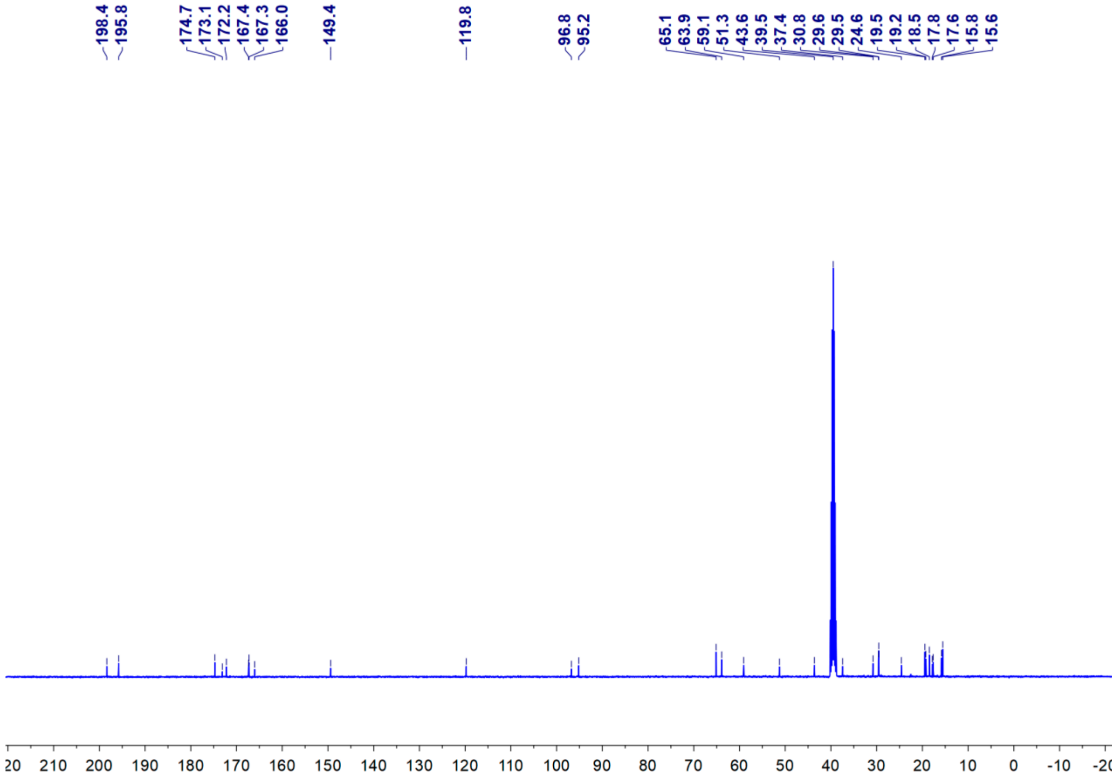

Fig. S46. ^{1}H-^{1}H **COSY spectrum (DMSO-*d6*, 400Hz) of 7**

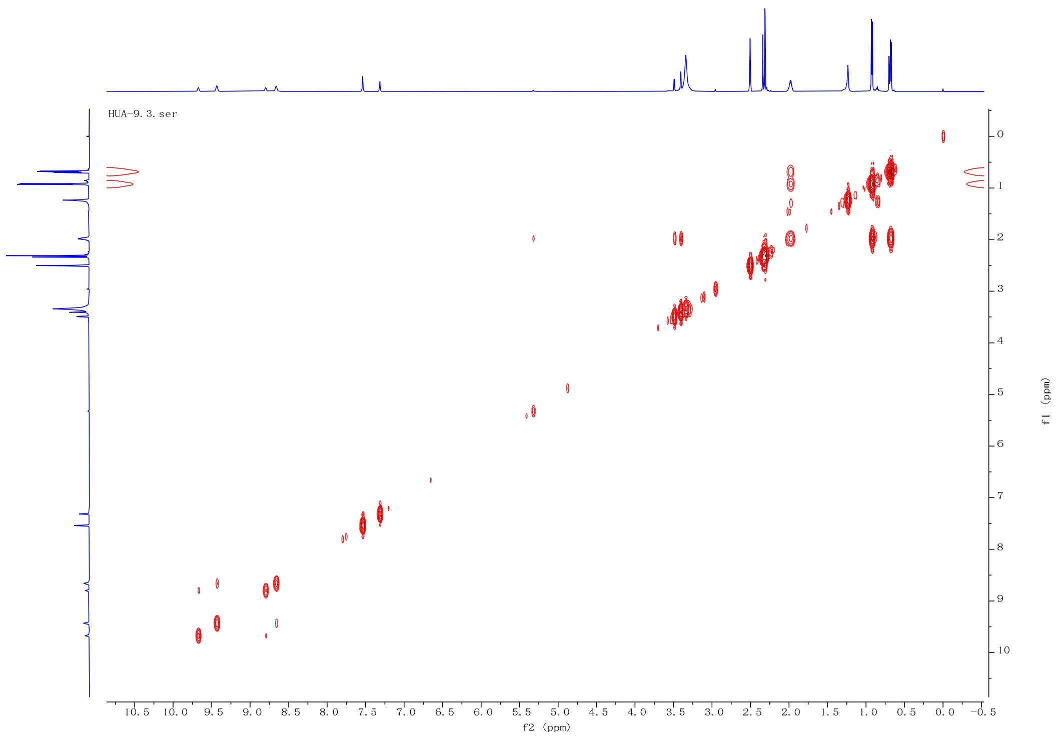

Fig. S47. HMBC spectrum (DMSO-*d6*, 400Hz) of 7

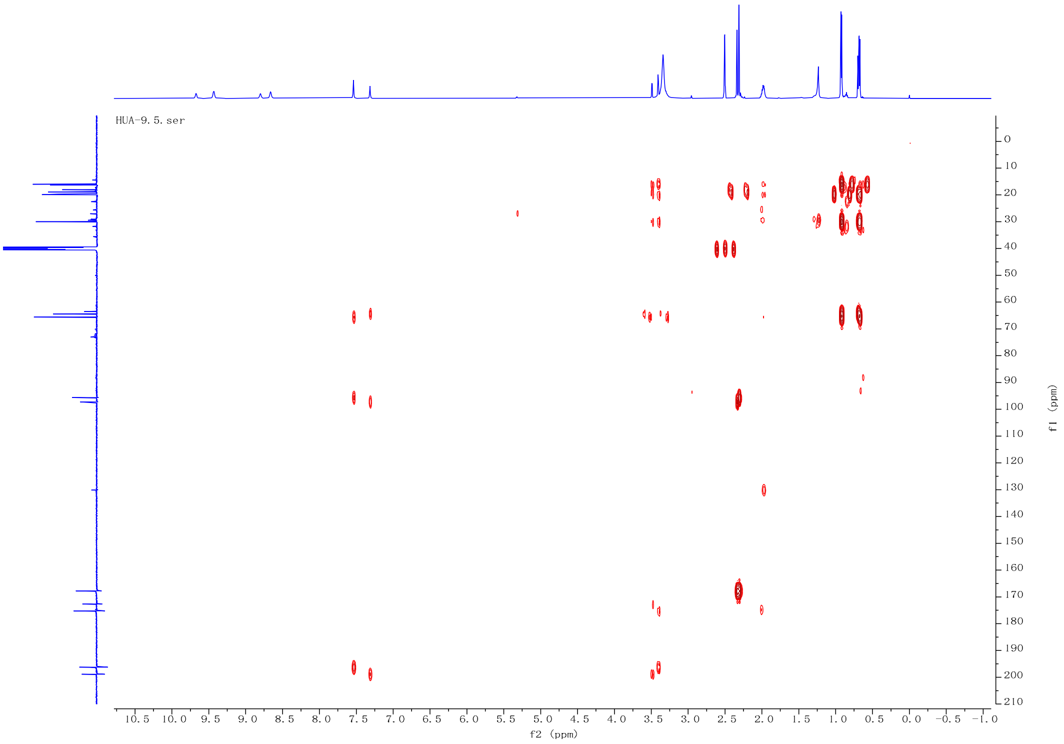

Fig. S48. HSQC spectrum (DMSO-*d6*, 400Hz) of 7

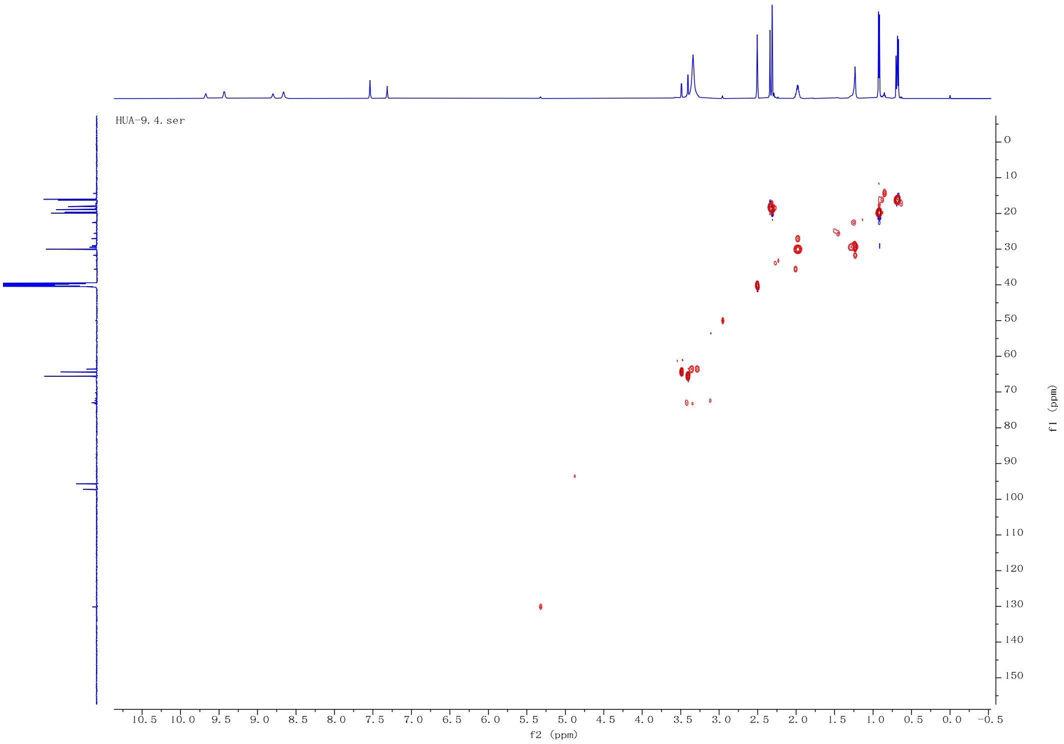

Fig. S49. UV spectrum (MeOH) of 7

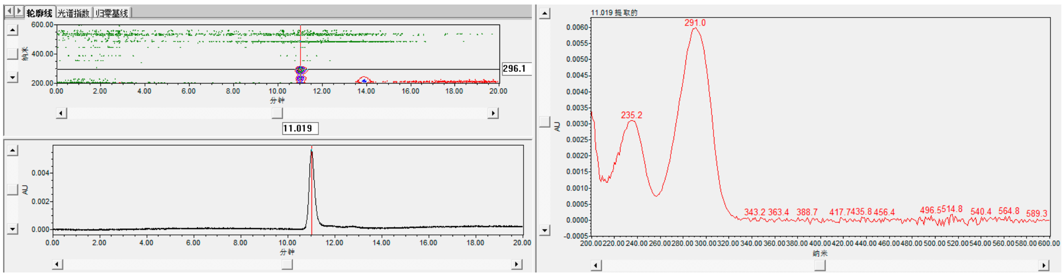

Fig. S50 (+)HR-ESI-MS of compound 7

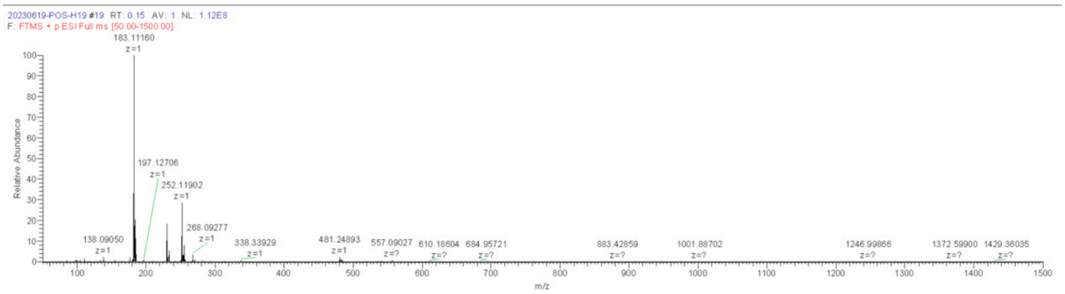

Fig. S51. ^{1}H NMR spectrum (DMSO-*d6*, 600Hz) of 8

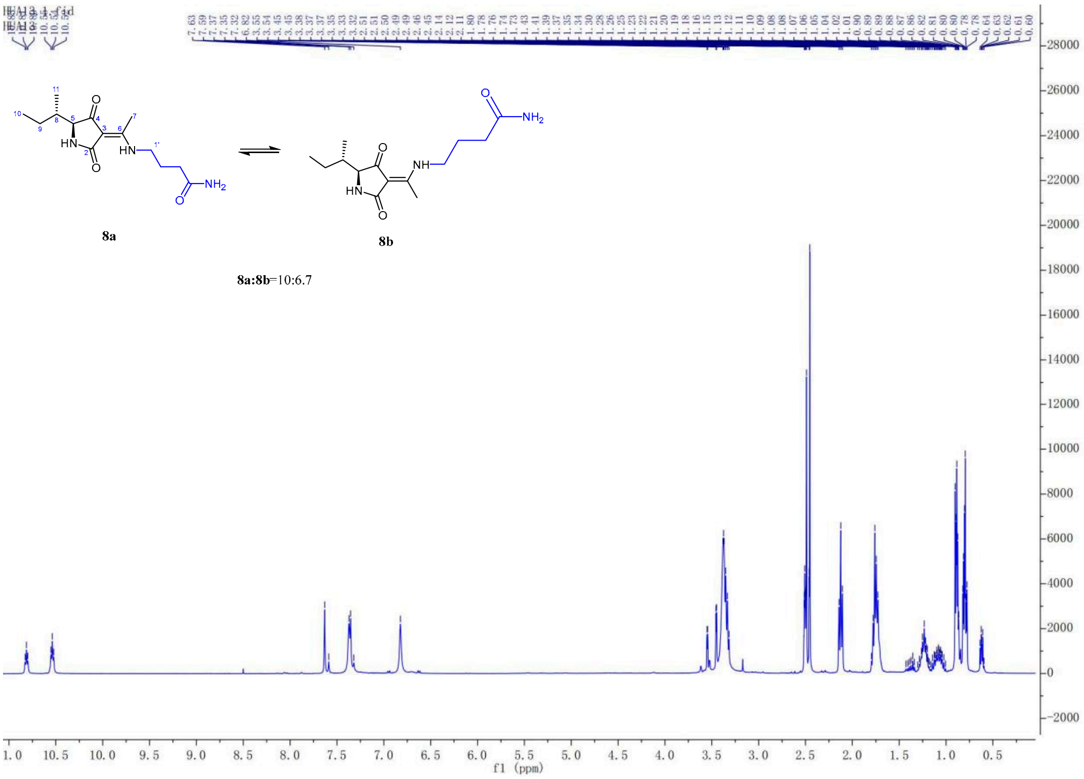

Fig. S52. ^{13}C NMR spectrum (DMSO-*d6*, 600Hz) of 8

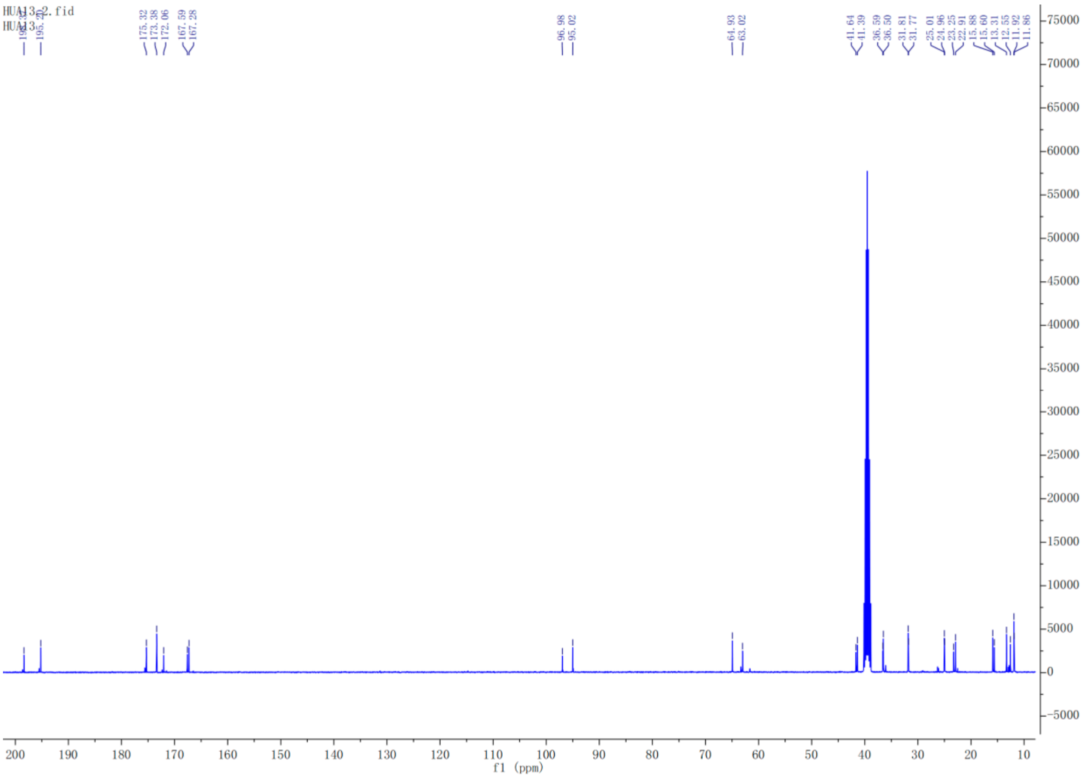

Fig. S53. ^{1}H-^{1}H COSY spectrum (DMSO-*d6*, 600Hz) of 8

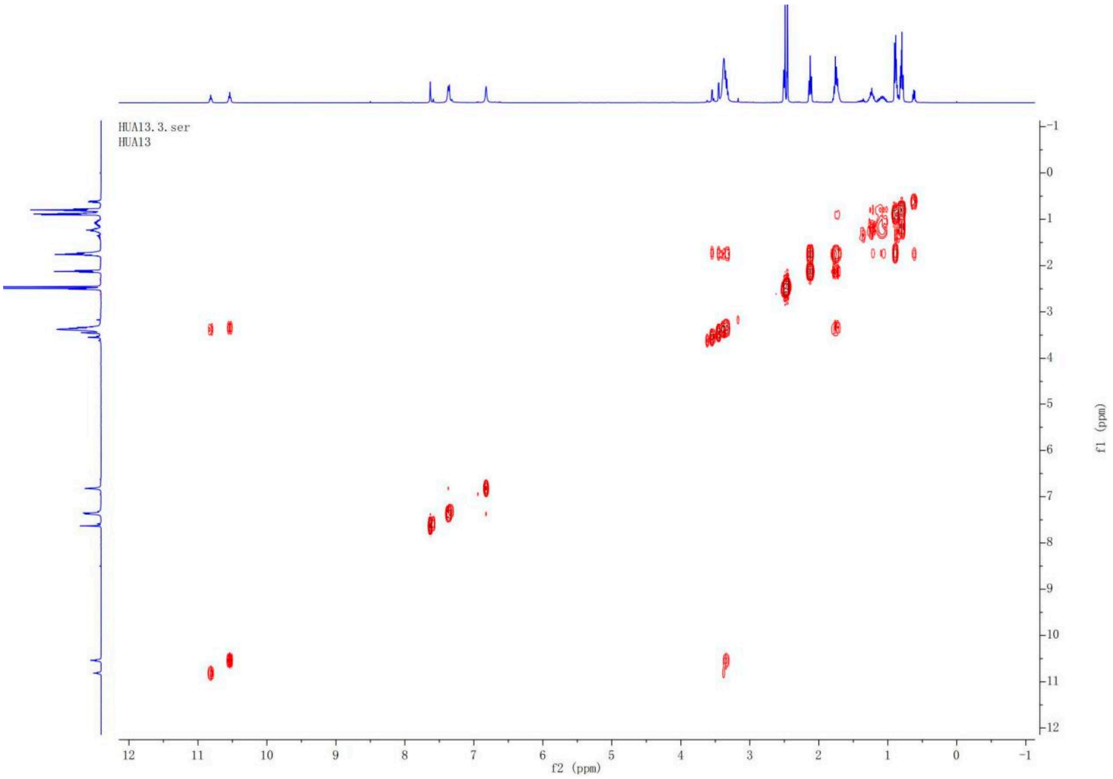

Fig. S54. HMBC spectrum (DMSO-*d6*, 600Hz) of 8

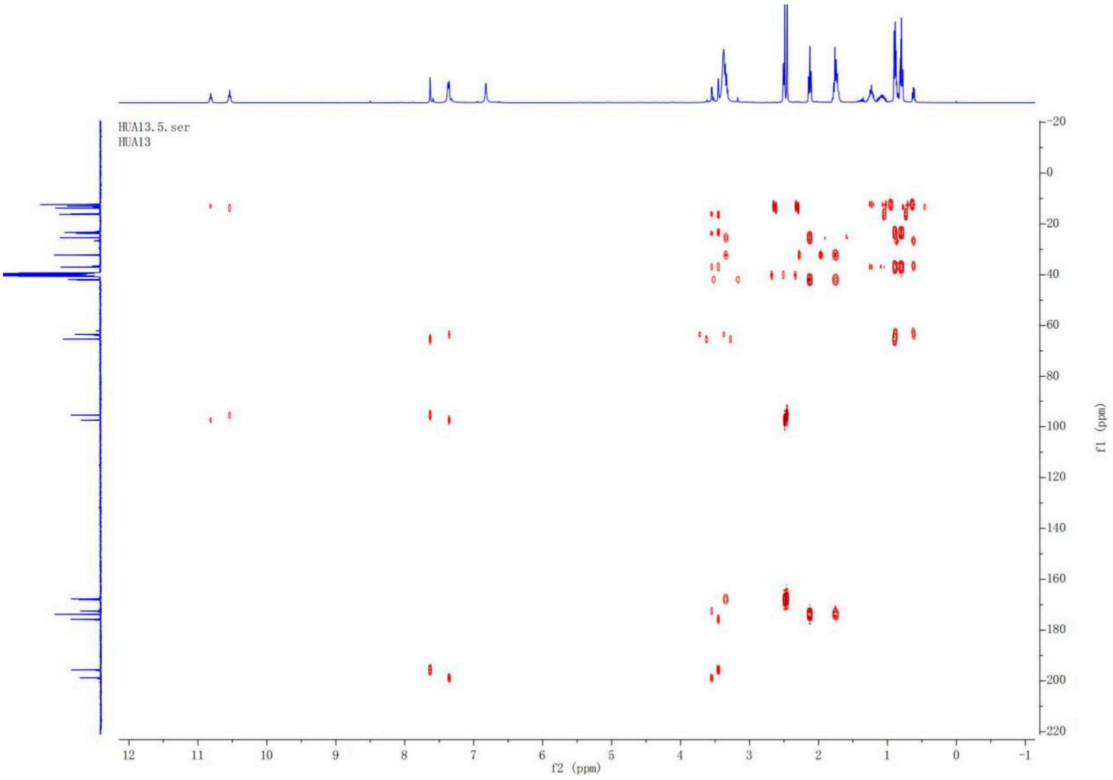

Fig. S55. HSQC spectrum (DMSO-*d6*, 600Hz) of 8

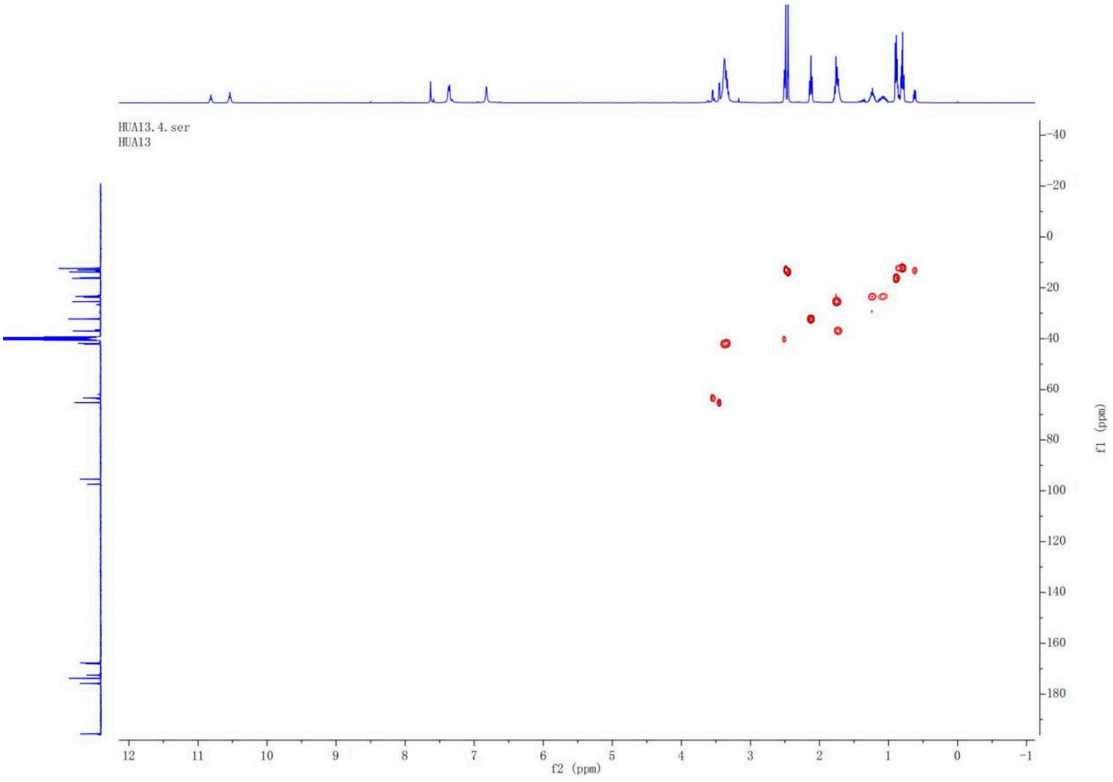

Fig. S56. (+)-HR-ESI-MS of compound 8

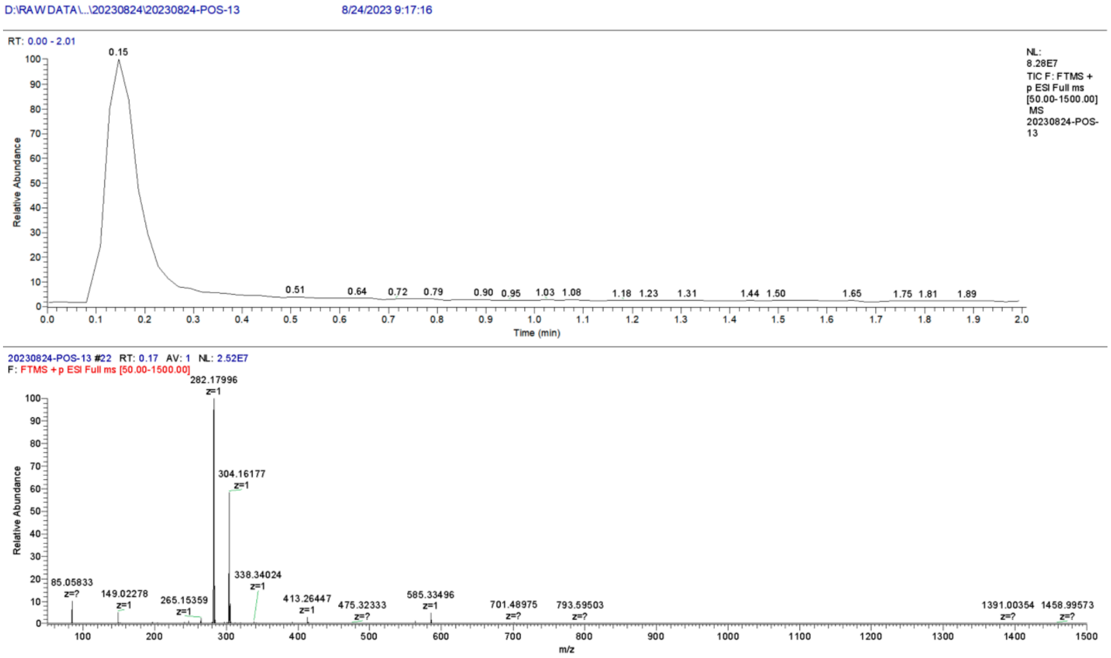

Supplement: Supplementary file 1 [file jof-10-00809-s001.zip › jof-3251779-supplementary.pdf]
